# Free tools for crystallographic symmetry handling and visualization

**DOI:** 10.1107/S1600576724007659

**Published:** 2024-09-20

**Authors:** Gemma de la Flor, Mois I. Aroyo, Ilaria Gimondi, Suzanna C. Ward, Koichi Momma, Robert M. Hanson, Leopoldo Suescun

**Affiliations:** a Institute of Applied Geosciences Karlsruhe Institute of Technology Karlsruhe Germany; b Departamento de Física Universidad del País Vasco UPV/EHU Spain; c Cambridge Crystallographic Data Centre United Kingdom; d National Museum of Nature and Science Tokyo Japan; e Department of Chemistry St Olaf College Northfield Minnesota USA; f Cryssmat-Lab/DETEMA, Facultad de Química Universidad de la República Montevideo Uruguay; Wilfrid Laurier University, Waterloo, Ontario, Canada

**Keywords:** symmetry visualization, *VESTA*, *Jmol*/*JSmol*, Cambridge Structural Database, Bilbao Crystallographic Server

## Abstract

Online courses and innovative teaching methods have triggered a trend in education, where the integration of multimedia, online resources and interactive tools is reshaping the view of both virtual and traditional classrooms. The use of interactive tools extends beyond the boundaries of the physical classroom, offering students the flexibility to access materials at their own speed and convenience and enhancing their learning experience. In the field of crystallography, there are a wide variety of free online resources such as web pages, interactive applets, databases and programs that can be implemented in fundamental crystallography courses for different academic levels and curricula. This paper discusses a variety of resources that can be helpful for crystallographic symmetry handling and visualization, discussing four specific resources in detail: the Bilbao Crystallographic Server, the Cambridge Structural Database, *VESTA* and *Jmol*. The utility of these resources is explained and shown by several illustrative examples.

## Abstract 2

In crystallography there exist a wide variety of online resources such as web pages, interactive applets, databases and programs that can be implemented in both virtual and traditional classrooms. Among these, the Bilbao Crystallographic Server, the Cambridge Structural Database, *Mercury*, *VESTA* and *Jmol* are highlighted for teaching fundamental crystallography since they are useful resources for crystallographic symmetry handling and visualization.

## 1. Introduction

The pandemic of 2020 caused a sudden change in every aspect of our lives, transforming how we interact and socialize with others, as well as revolutionizing our way of working. The field of education was not exempt from this change, as educators were forced to transition their classes from in-person to remote learning. This radical change was a great challenge at all academic levels, since lecturers had to find ways to not only keep the classes running during lockdown but also still inspire and engage their students. One of the problems that many lecturers had to face was to adapt the traditional resources used in physical classrooms to the virtual learning environment. Many of the tools and materials necessary to perform this transition were already developed, but lecturers were either not aware of these resources or not familiar with them. In the field of fundamental crystallography, for example, there exist a wide variety of free online resources such as web pages, interactive applets, databases and programs that can be used in virtual classrooms as well as in-person ones. Many of these are useful for crystallographic symmetry handling and visualization.

Basic fundamental crystallography courses usually start by introducing the concept of symmetry in crystallography. Students not only have to gain insight into the different types of symmetry exhibited by macroscopic crystals but also need to acquire the skills to identify them and determine their corresponding crystallographic point group. Traditionally, these competences were acquired by analysing the symmetry of classical crystallographic wooden models or molecular models. In an online classroom, however, it is necessary to replace the physical models used in a traditional classroom by virtual ones. The *interactive PDF files with embedded 3D models* (Arribas *et al.*, 2014 ▸), inspired by classical wooden crystallographic models, visualize for each of the 32 crystallographic point groups interactive three-dimensional polyhedra (used to represent idealized crystals), their cor­responding symmetry elements and the stereographic projection. This material is freely available online (https://github.com/LluisCasas/GSP) and the resource can be very helpful for students to gain a solid grasp of the concepts of symmetry and point groups. With a more atom-based approach, *Jmol* (Hanson, 2013 ▸) allows the visualization of three-dimensional representations of molecules and crystal structures that can also be used in a classroom to analyse the symmetry and determine the point group of a given molecule (https://chemapps.stolaf.edu/jmol/jsmol/jpge/). In addition, the program offers the capability of visually demonstrating the action of the symmetry operations in the molecule, helping develop students’ understanding of these concepts.

Once students have a solid understanding of point groups, the next step is to introduce the concept of translational symmetry followed by the definition of space groups. This knowledge is essential to describe the atomic structure of three-dimensional crystals. Besides *Jmol*, the freely available program *VESTA* (https://jp-minerals.org/vesta/en/; Momma & Izumi, 2011 ▸) can also be used for visualizing three-dimensional crystal structures. Apart from crystal structural models, this program can be applied to visualize lattice planes and crystal morphologies. The programs *Jmol* and *VESTA* go beyond the simple visualization of crystal structures from uploaded crystallographic files. They allow the user to construct crystal structural models step by step, first building the unit cell by specifying the space group and lattice parameters, and then adding the atoms into the empty unit cell. This feature is valuable for students since it provides a comprehensive learning experience.

The use and understanding of the crystallographic data on space groups in *International Tables for Crystallography* (2016 ▸), Vol. A, *Space-Group Symmetry* (henceforth abbreviated as *IT*A), is also an important part of the curriculum for graduate students. A significant number of students, however, lack access to either the printed or online editions of *IT*A. Fortunately, there are free resources on the web that contain essential information about space groups, such as the Bilbao Crystallographic Server (https://www.cryst.ehu.es/; Aroyo *et al.*, 2011 ▸; Tasci *et al.*, 2012 ▸; hereafter referred to as BCS) and the *Hypertext Book of Crystallographic Space Group Diagramsand Tables* (http://img.chem.ucl.ac.uk/sgp/mainmenu.htm). In addition to granting access to their extensive database, the BCS offers more complex programs to study problems in crystallography, such as the comparison of structures, crystal structure data transformation or phase transitions – topics that are often part of the graduate-level curriculum. Recent developments in *Jmol* allow the visualization of space-group symmetry (https://djohnston66.gitlab.io/sgexplorer/). The availability of such databases and programs can enhance these students’ learning experience and capabilities.

Crystal structure databases also play an important role in the teaching of crystallography, providing real-world examples that allow students to apply theoretical concepts in a practical context. This practical approach can improve comprehension and reinforce their grasp of crystallographic principles. By exploring actual crystal structures, students gain insights into symmetry, atomic arrangements and other key principles. This allows a visual understanding of the concepts introduced in their studies. Depending on the discipline, there are many online free-of-charge databases, including the Protein Data Bank (https://www.rcsb.org/), Crystallography Open Database (Gražulis *et al.*, 2009 ▸; https://www.crystallography.net/cod/), AFLOW Encyclopaedia of Crystal Structure Prototypes (Mehl *et al.*, 2017 ▸; https://aflowlib.org/prototype-encyclopedia/), Magnetic Structure Database (Gallego *et al.* 2016*a* ▸,*b* ▸; https://www.cryst.ehu.es/magndata/), Bilbao Incommensurate Crystal Structure Database (https://www.cryst.ehu.eus/bincstrdb/), and the joint Cambridge Crystallographic Data Centre (CCDC) and FIZ Karlsruhe Access Structures Service (https://www.ccdc.cam.ac.uk/structures) which provides access to data sets in the Cambridge Structural Database (CSD; Groom *et al.*, 2016 ▸) and the Inorganic Crystal Structure Database (ICSD; Zagorac *et al.*, 2019 ▸). The CCDC also offers the *Mercury* visualization tool (Macrae *et al.*, 2020 ▸), allowing for the building of structural models based on symmetry-related units, drawing symmetry elements within the unit cell, and click calculation of interatomic distances and angles.

In this paper we describe four freely available tools that can be used in fundamental crystallography courses for bachelor’s and master’s curricula focused on symmetry handling and visualization: the BCS, the CSD and its associated *Mercury* program, *VESTA*, and *Jmol*. The utility of these tools will be shown using practical examples.

## 2. Crystallographic databases and programs hosted at the Bilbao Crystallographic Server

Operating since 1998, the BCS is a free web server that grants access to crystallographic databases and programs to resolve different types of problems related to crystallography, crystal chemistry, solid-state physics and materials science. The programs available on the server do not require a local installation and their use is free of charge. The server is built on a core of databases that includes data from *IT*A, *Inter­national Tables for Crystallography*, Vol. A1, *Symmetry Relations Between Space Groups* (2010*a* ▸) (henceforth abbreviated as *IT*A1) and *International Tables for Crystallography*, Vol. E, *Subperiodic Groups* (2010*b* ▸). Therefore, the server gives access to crystallographic data on space, subperiodic, plane and point groups. In addition to this, there is access to the normalizers of space groups database which contains data on the Euclidean, chirality-preserving Euclidean and affine normalizers of the space groups. A **k**-vector database with Brillouin-zone figures and classification tables of all the wavevectors for all 230 space groups (Aroyo *et al.*, 2014 ▸) and 80 layer groups (de la Flor *et al.*, 2021 ▸) is also available, together with the magnetic (Perez-Mato *et al.*, 2015 ▸) and double-space-group databases (Elcoro *et al.*, 2017 ▸). The BCS also hosts the magnetic and incommensurate structure databases.

The main aim of the BCS is to bring the potential of group theory to those users who are not experts in this matter but nevertheless want to use it in their research. In addition, the server is a valuable resource for teaching crystallography to students across different academic levels, from bachelor’s to PhD. This dual functionality highlights its versatility in both research and educational domains.

### 2.1. Crystallographic space-group databases

Most of the space-group data compiled in *IT*A, such as the generators, general positions, Wyckoff positions, geometric interpretation of symmetry operations, reflection conditions and normalizers, are available in the *Space-group symmetry* section of the BCS. These data can usually be retrieved by specifying the space-group number; if the number is unknown, the space group can be selected from a table with the Hermann–Mauguin symbols. The databases and programs of the BCS use specific settings of space groups, termed *standard* or *default* settings. These are settings that coincide with the *conventional* ones.^1^ For space groups with more than one conventional description in *IT*A, the following settings are chosen as standard: (i) *unique axis b setting*, *cell choice 1* for monoclinic groups, (ii) *hexagonal axes setting* for rhombohedral groups, and (iii) *origin choice 2* description for the centrosymmetric groups listed with respect to two origins in *IT*A (*i.e.* when the origin is at a centre of inversion). The BCS hosts data for all 230 space groups in their default settings, and in addition the crystallographic data of the settings for the monoclinic and orthorhombic space groups listed in Table 1.5.4.4 of *IT*A (henceforth abbreviated as *IT*A settings^2^). Some of the programs available in the BCS and described in the following sections can also be found in the *Symmetry Database* (de la Flor *et al.*, 2023 ▸; https://symmdb.iucr.org/), available only to subscribers of the online version of *International Tables for Crystallography* (https://it.iucr.org/).

#### 2.1.1. Generators and general position of space groups

The generators and general position of space groups are shown by the program *GENPOS*. The generators and general position entries are specified by their coordinate triplets, the matrix–column representations of the corresponding symmetry operations and their geometric interpretations.

(i) The list of coordinate triplets (*x*, *y*, *z*) reproduces the data of the *General position* blocks of space groups found in *IT*A. The coordinate triplets may also be interpreted as shorthand descriptions of the matrix presentation of the corresponding symmetry operations.

(ii) The matrix–column presentation of symmetry operations is defined as follows. With reference to a coordinate system consisting of an origin *O* and a basis (**a**_1_, **a**_2_, **a**_3_), the symmetry operations of space groups are described by (3 × 4) matrix–column pairs (**W**, **w**).

(iii) The geometric interpretation of symmetry operations is given (*a*) following the conventions in *IT*A [including the symbol of the symmetry operation, its glide or screw component (if relevant), and the location of the related geometric element] and (*b*) using the Seitz notation [see Glazer *et al.* (2014 ▸)].

**Figure 1:**
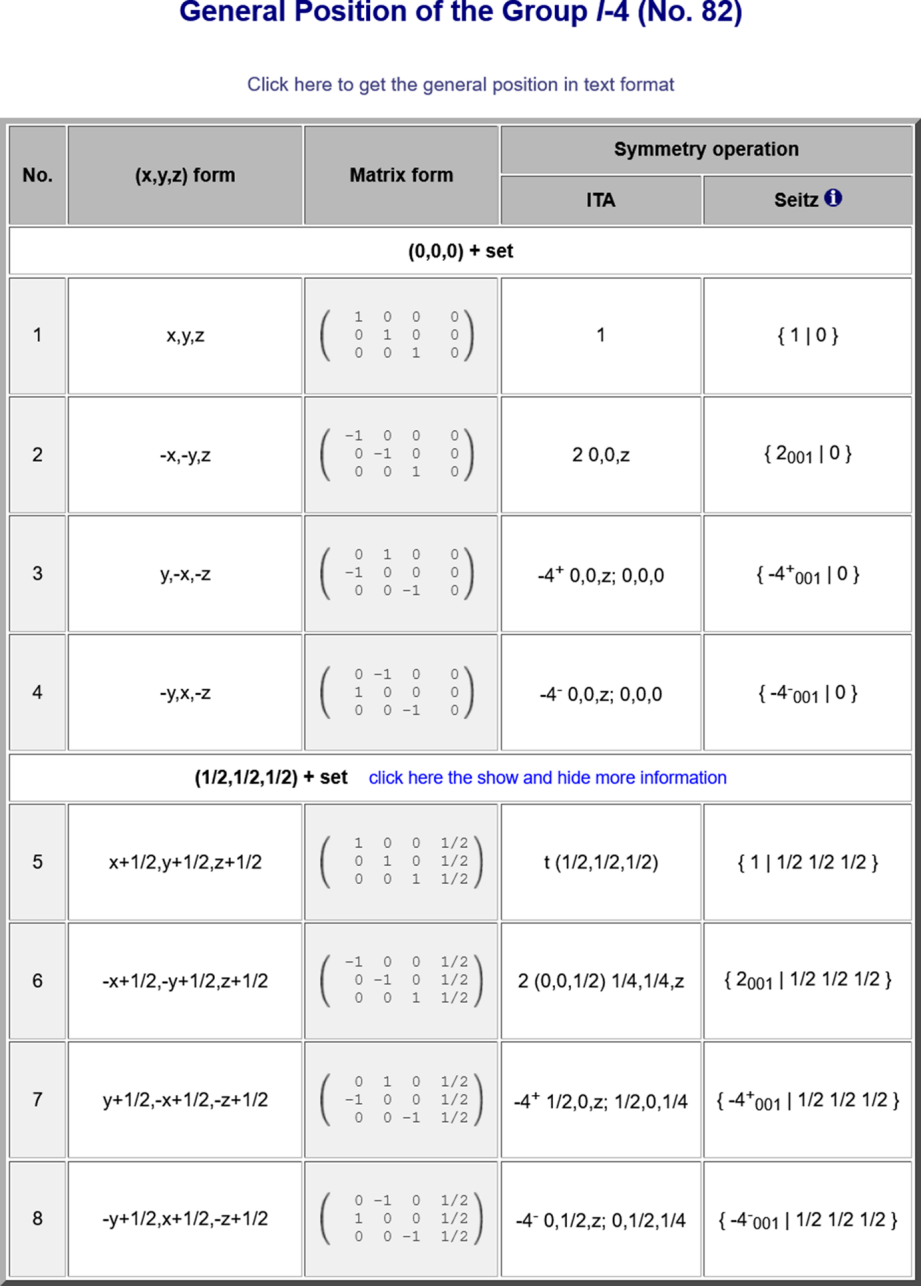
The general position of the space group 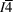 (No. 82) in the standard setting given by the program *GENPOS*.

For space groups with a centred lattice, the general position is shown in blocks. The number of blocks is equal to the multiplicity of the centred cell. Fig. 1 ▸ shows the general position for the space group 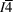 (No. 82) in the standard setting. In this particular example, there are two blocks of general position, one for the ‘(0,0,0)+ set’ and the other for the ‘(1/2,1/2,1/2)+ set’.

The program *GENPOS* lists the generators and general position entries of space groups in the standard setting (option *Standard/Default Setting*) as well as in one of the *IT*A settings. On clicking on the option *ITA Settings*, a list of the *IT*A settings of the chosen space-group settings is shown; the corresponding data can be calculated with respect to one of these settings by choosing it directly from this list. The general position of the selected setting is listed in the same way as in Fig. 1 ▸, together with the corresponding listing for the standard setting of the space group.

In addition, the program can produce the data in any other non-standard setting via the option *User-Defined Setting*, provided the transformation relating the origin and basis of the non-standard setting to those of the standard setting is specified. The coordinate transformation is described by a matrix–column pair (**P**, **p**) and consists of two parts: (i) a linear part **P** defined by a (3 × 3) matrix, which describes the change in direction and/or length of the basis vectors (**a**_1_, **a**_2_, **a**_3_)_non-stand_ = (**a**_1_, **a**_2_, **a**_3_)_stand_**P** [where (**a**_1_, **a**_2_, **a**_3_)_non-stand_ is the basis of the non-standard setting and (**a**_1_, **a**_2_, **a**_3_)_stand_ is the basis of the standard setting], and (ii) an origin shift **p** = (*p*_1_, *p*_2_, *p*_3_)^T^ defined as a (3 × 1) column matrix, whose coefficients describe the position of the non-standard origin with respect to the standard one. The data of the matrix–column pair 
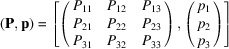
are often written in the following concise form: 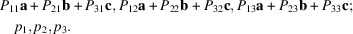
The program *GENPOS* allows the data to be transformed to any setting.

#### 2.1.2. Wyckoff positions of space groups

**Figure 2:**
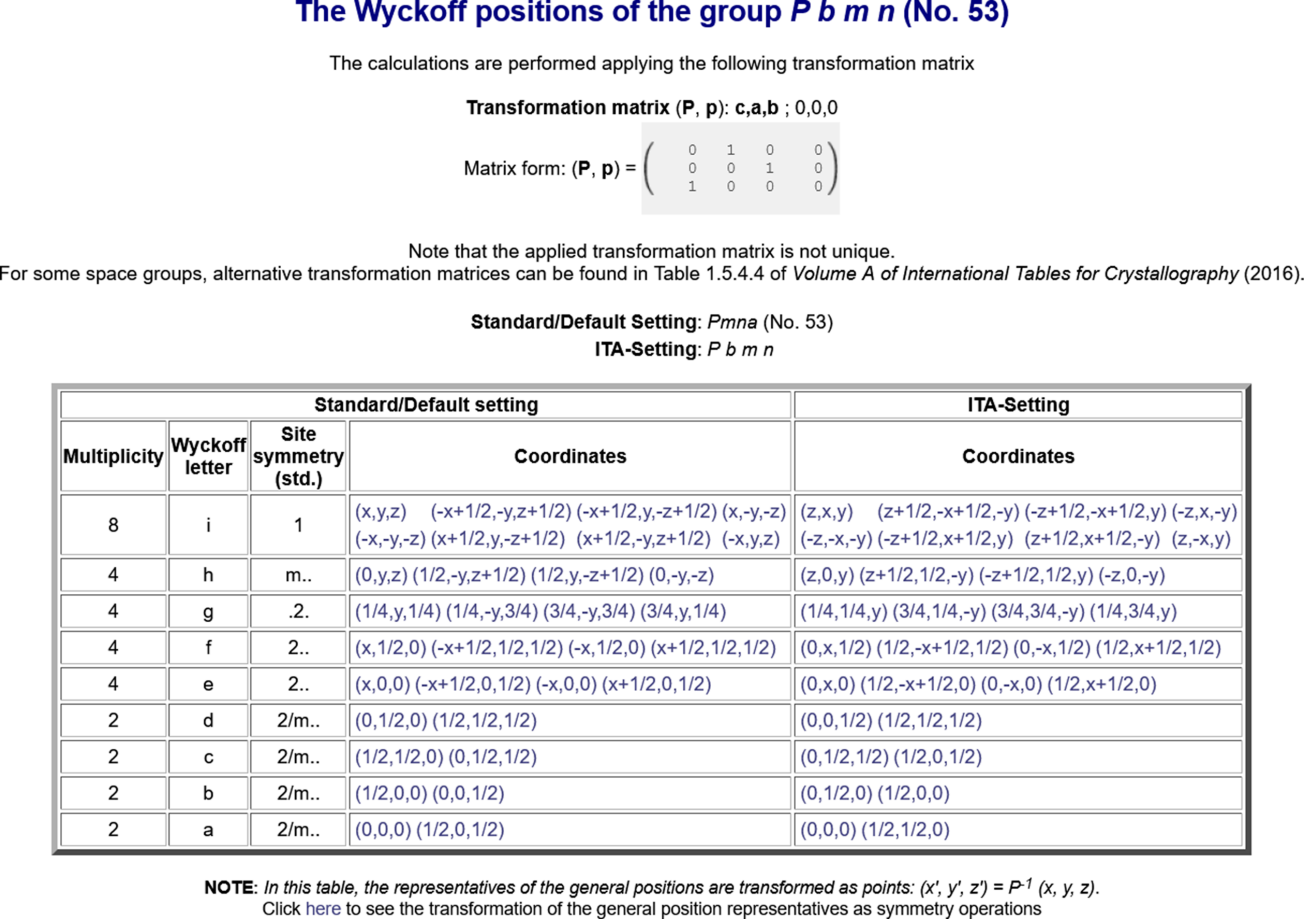
The Wyckoff positions of the space group *Pmna* (No. 53) with respect to the standard setting and its *IT*A setting *Pbmn*, as displayed by the program *WYCKPOS* with the option *ITA Settings*.

The program *WYCKPOS* provides a list of Wyckoff positions, in the standard setting (option *Standard/Default Setting*) as well as in different settings, for a designated space group. The listing follows that of *IT*A (*cf.* Fig. 2 ▸): the Wyckoff-position block starts with the general-position data at the top, followed downwards by various special Wyckoff positions with decreasing multiplicity and increasing site symmetry. The data for each Wyckoff position include (i) the multiplicity, *i.e.* the number of equivalent positions in the conventional unit cell; (ii) the Wyckoff letter, which is an alphabetical label; (iii) the site symmetry group; and (iv) a set of coordinate triplets of the equivalent Wyckoff position points in the unit cell, shown under the column ‘Coordinates’. For space groups with a centred lattice, the centring translations are listed above the coordinate triplets. The point group isomorphic to the site-symmetry group is indicated by an oriented symbol (column three of Fig. 2 ▸), which is a variation of the Hermann–Mauguin point-group symbol that provides information about the orientation of the symmetry elements (for more details, see Section 2.1.3.12 of *IT*A). An explicit listing of the symmetry operations of the site-symmetry group of a point is obtained by clicking directly on its coordinate triplet. Optionally, the symmetry operations of the site-symmetry groups of an arbitrary point (specified by its coordinates but not necessarily within the unit cell) can be calculated using the auxiliary tool below the Wyckoff position table.

Apart from the *Standard/Default setting* option, the program is also able to calculate the Wyckoff positions in different space-group settings, either by specifying the co­ordinate transformation (**P**, **p**) to a new basis (option *User-Defined setting*) or by selecting directly one of the *IT*A settings of the corresponding space group (option *ITA Settings*), thus enhancing and extending the data in *IT*A.

#### 2.1.3. Geometric interpretation of matrix–column representations (**W**, **w**) of symmetry operations

Analysing the rotational part **W** and the translational part **w** of the matrix–column representation (**W**, **w**) of symmetry operations, it is possible to calculate their corresponding geometric interpretation if the coordinate system to which (**W**, **w**) refers is known [see Section 1.2.2.4. of *IT*A, and Stróż (2007 ▸)]. The geometric interpretation of symmetry operations can be independently calculated by the program *SYMMETRY OPERATIONS* of the BCS.

**Figure 3:**
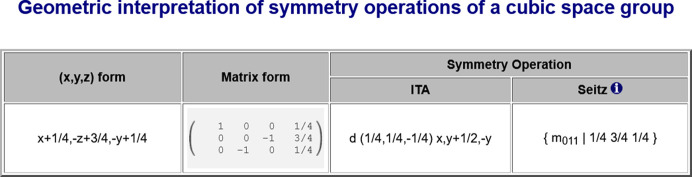
A geometric interpretation of the symmetry operation 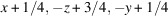, described with respect to the standard setting of a space group belonging to the cubic crystal system, as determined by the program *SYMMETRY OPERATIONS*. It corresponds to a *d*-glide reflection through the plane (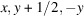) and a glide vector with components (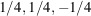).

The input of this program consists of the space-group number or the crystal system, and the matrix–column representation of the symmetry operation, which can be given by its coordinate triplet or in matrix form. With the option *Standard/Default Setting* the program returns a table with the symmetry operation described by its coordinate triplet, matrix–column pair presentation, and geometric interpretation following the conventions of *IT*A and using the Seitz notation. As an example, Fig. 3 ▸ shows the geometric interpretation of the symmetry operation 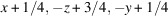 described with respect to the standard setting and belonging to the cubic crystal system calculated by the program *SYMMETRY OPERATIONS*. The program also allows these calculations in *IT*A settings, using the option *ITA Settings*, and in any other non-standard setting by using the option *User-Defined Setting*.

### 2.2. Group–subgroup relations of space groups

The data compiled in *IT*A1, such as the maximal subgroup of space groups, the minimal supergroups of space groups, and the relationships between the Wyckoff positions of space groups and their subgroups, are available in the sections *Space-group symmetry* and *Group–subgroup relations of space groups* of the BCS. The data in *IT*A1 on maximal subgroups of space groups of indices 2, 3 and 4 are extended to include the series of all isomorphic subgroups for indices up to 27 (125 for some cubic groups). In contrast to *IT*A1, where only space-group types of minimal supergroups are indicated, the database of the BCS contains individual information for each minimal supergroup, including the transformation matrix that relates the conventional bases of the group and the minimal supergroup.

#### 2.2.1. Maximal subgroups of space groups

**Figure 4:**
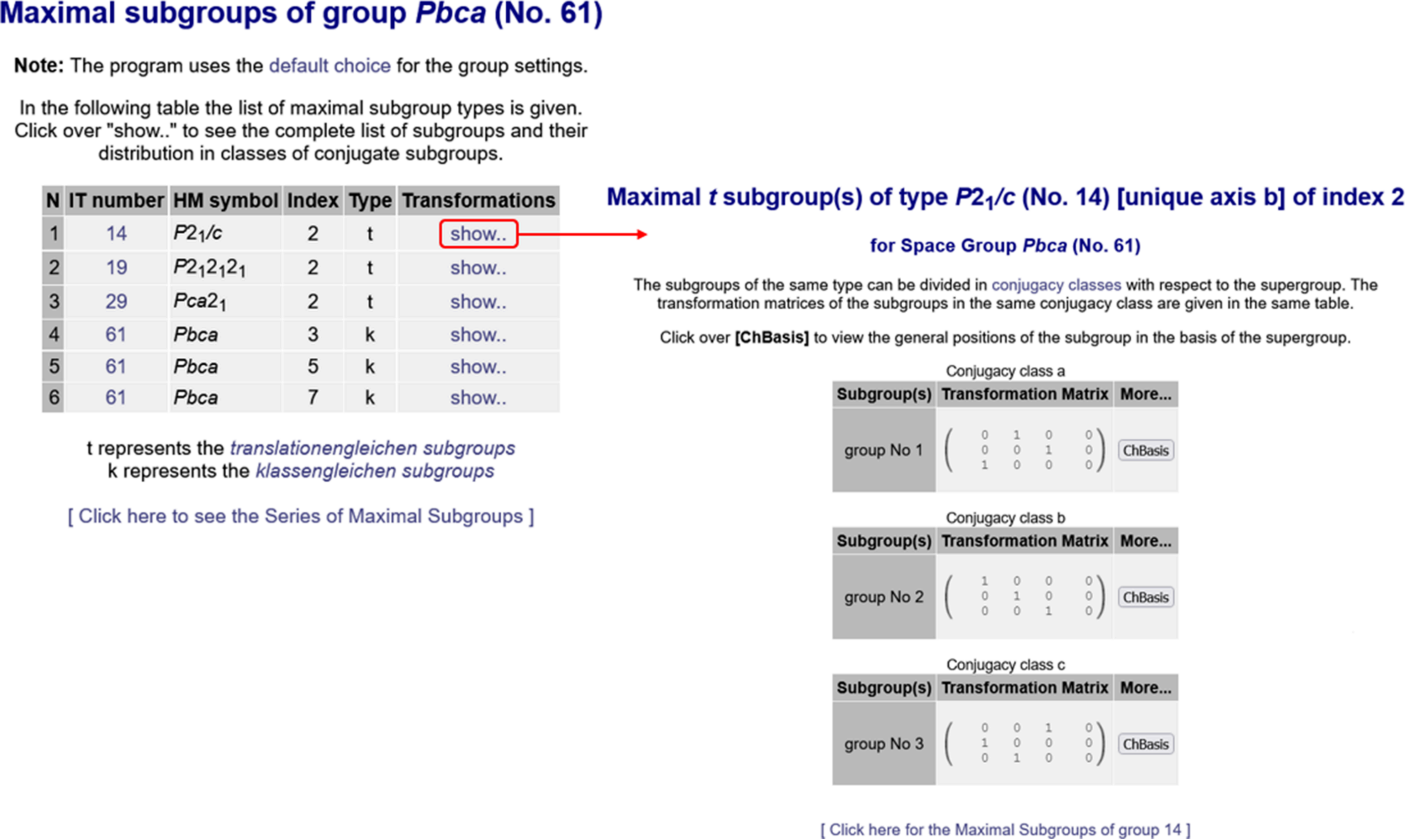
A list of the maximal subgroups of the space group *Pbca* (No. 61) up to index 7, as displayed by the program *MAXSUB*. A click on *show..* reveals that there are three different subgroups of the type *P*2_1_/*c* (No. 14) of index 2, each of them specified by different transformation matrices, as shown on the right-hand side.

The program *MAXSUB* gives access to the database of maximal subgroups of space groups. For a designated space group, *MAXSUB* lists the maximal subgroups of indices up to index 9. This program first returns a table with the maximal subgroup types of the selected space group (left-hand table in Fig. 4 ▸). Each subgroup type is specified by its space-group number, Hermann–Mauguin symbol, index and subgroup type (*t* for *translationen­gleiche* and *k* for *klassengleiche* or *isomorphic*). The complete list of subgroups and their distribution in classes of conjugate subgroups is obtained by clicking on the link *show..* . For example, the space group *Pbca* (No. 61) has three maximal *translationengleiche* subgroups of the type *P*2_1_/*c* (No. 14) of index 2 (right-hand table in Fig. 4 ▸). The transformation matrix–column pairs (**P**, **p**) that relate the standard bases of the subgroup and the group are also provided by the program. For certain applications, it is necessary to transform the general-position representatives of the subgroup by the corresponding matrix–column pair (**P**, **p**)^−1^ to the coordinate system of the group by the option *ChBasis*. The relations between the Wyckoff positions of the space group and those of its maximal subgroups can also be calculated by *MAXSUB*, provided the option *Show WP Splittings* is selected in the input page of the program.

Every space group has an infinite number of maximal isomorphic subgroups of indices of *p*, *p*^2^ or *p*^3^ (where *p* is a prime), and these are distributed into several series of maximal isomorphic subgroups. In most of the series, the Hermann–Mauguin symbol for each isomorphic subgroup is the same. However, if the space group belongs to one of the 11 pairs of enantiomorphic space-group types, the Hermann–Mauguin symbol of an isomorphic subgroup is either that of the group or that of its enantiomorphic pair. The program *SERIES* provides access to the database of maximal isomorphic subgroups. Apart from parametric descriptions of the series, the program provides individual listings for all maximal isomorphic subgroups. For each series, the Hermann–Mauguin symbol of the subgroup, the restrictions on the parameters describing the series, and the transformation matrix relating the group and the subgroup are listed. There is also an option in the program that permits the online generation of maximal isomorphic subgroups of any allowed index. The format and content of the subgroup data are similar to those of the program *MAXSUB*.

#### 2.2.2. Splitting of the Wyckoff positions of space groups

**Figure 5:**
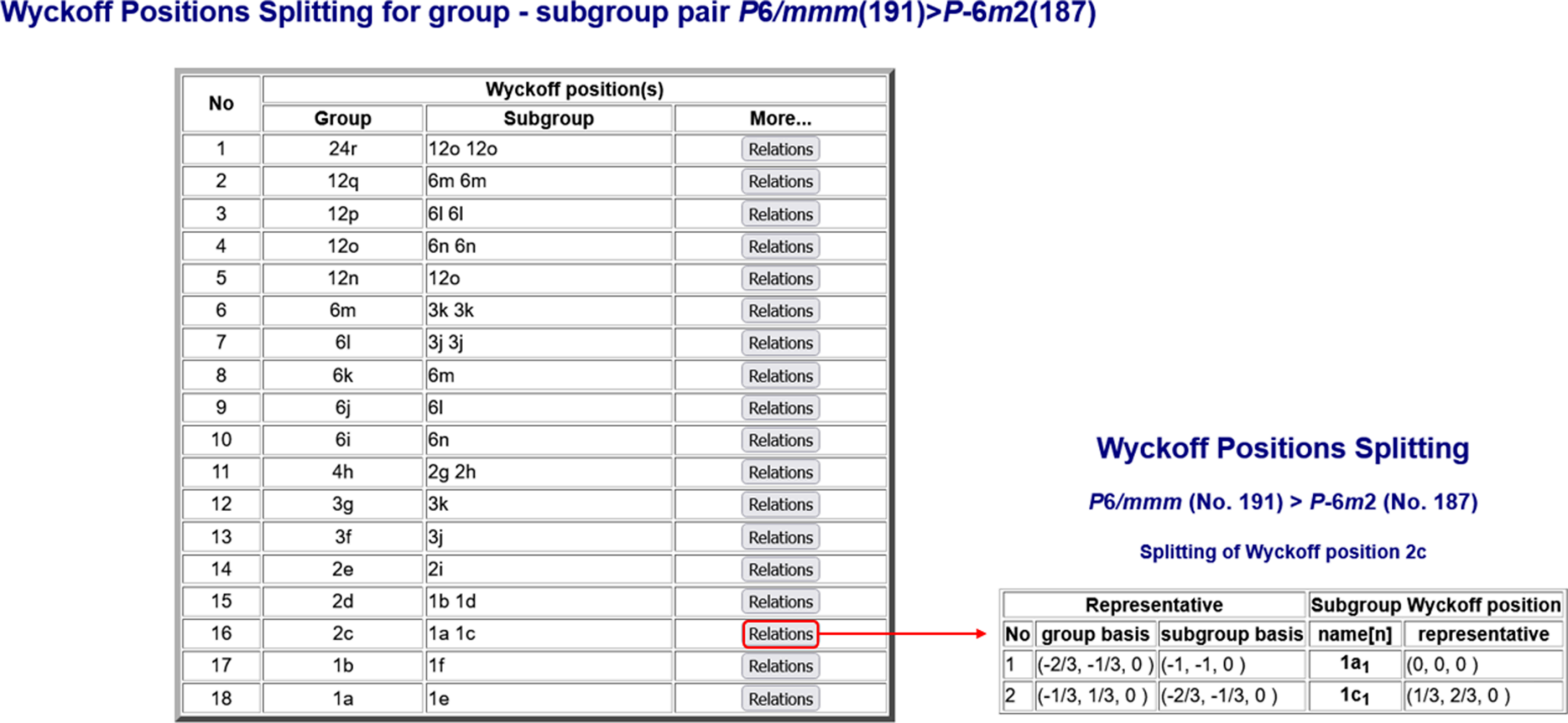
The Wyckoff-position splitting scheme between the space group *P*6/*mmm* (No. 191) and its maximal subgroup 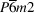 (No. 187) calculated by the program *WYCKSPLIT*. A click on *Relations* reveals the relations between the coordinate triplets of the Wyckoff position 2*c*, as shown on the right-hand side.

The program *WYCKSPLIT* (Kroumova *et al.*, 1998 ▸), available in the section *Group–subgroup relations of space groups* of the BCS, is designed to compute the relationships between the Wyckoff positions of a given space group and one of its subgroups. These relationships may involve splittings of the Wyckoff positions, reductions in the site symmetries or both. The program requires as input (i) the group and subgroup space-group numbers, (ii) the transformation matrix–column pair (**P**, **p**) that relates the basis of the group to that of the subgroup, and (iii) the Wyckoff positions of the group to be split. As output it returns the relationships between the Wyckoff positions of the group and those of the subgroup, these relationships being specified by their respective multiplicities and Wyckoff letters. The program *WYCKSPLIT* provides further information on the relations of the Wyckoff positions that are not listed in *IT*A1, namely the relations between the unit-cell representatives of the orbit of the group and the corresponding representatives of the suborbits of the subgroup. For example, the left-hand table in Fig. 5 ▸ shows the Wyckoff-position splitting schemes for the group–subgroup pair *P*6/*mmm* (No. 191) > 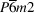 (No. 187), (**P**, **p**) = (**I**, **o**) [here **I** is the three-dimensional unit matrix and **o** is a (3 × 1) column matrix containing zeros as coefficients]. The Wyckoff position 2*c* of *P*6/*mmm* splits into two independent positions of 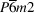 with no site-symmetry reduction,
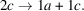
The relations between the coordinate triplets of the Wyckoff positions are displayed when clicking on *Relations*. These relations are presented in a table showing the Wyckoff-position coordinate triplets with respect to the standard group basis and the corresponding triplets referred to the basis of the subgroup (right-hand table in Fig. 5 ▸).

### 2.3. Structure Utilities

There is a section in the BCS entitled *Structure Utilities* which contains interesting programs for coordinate transformations, comparison of structures and the evaluation of structure relationships between group–subgroup-related structures. As input, these programs accept both standard crystallographic files in Crystallographic Information Framework (CIF) format and the server’s default structure definition format called *BCS format*; comments can be included by inserting a hash ‘#’ sign at the beginning of a line:
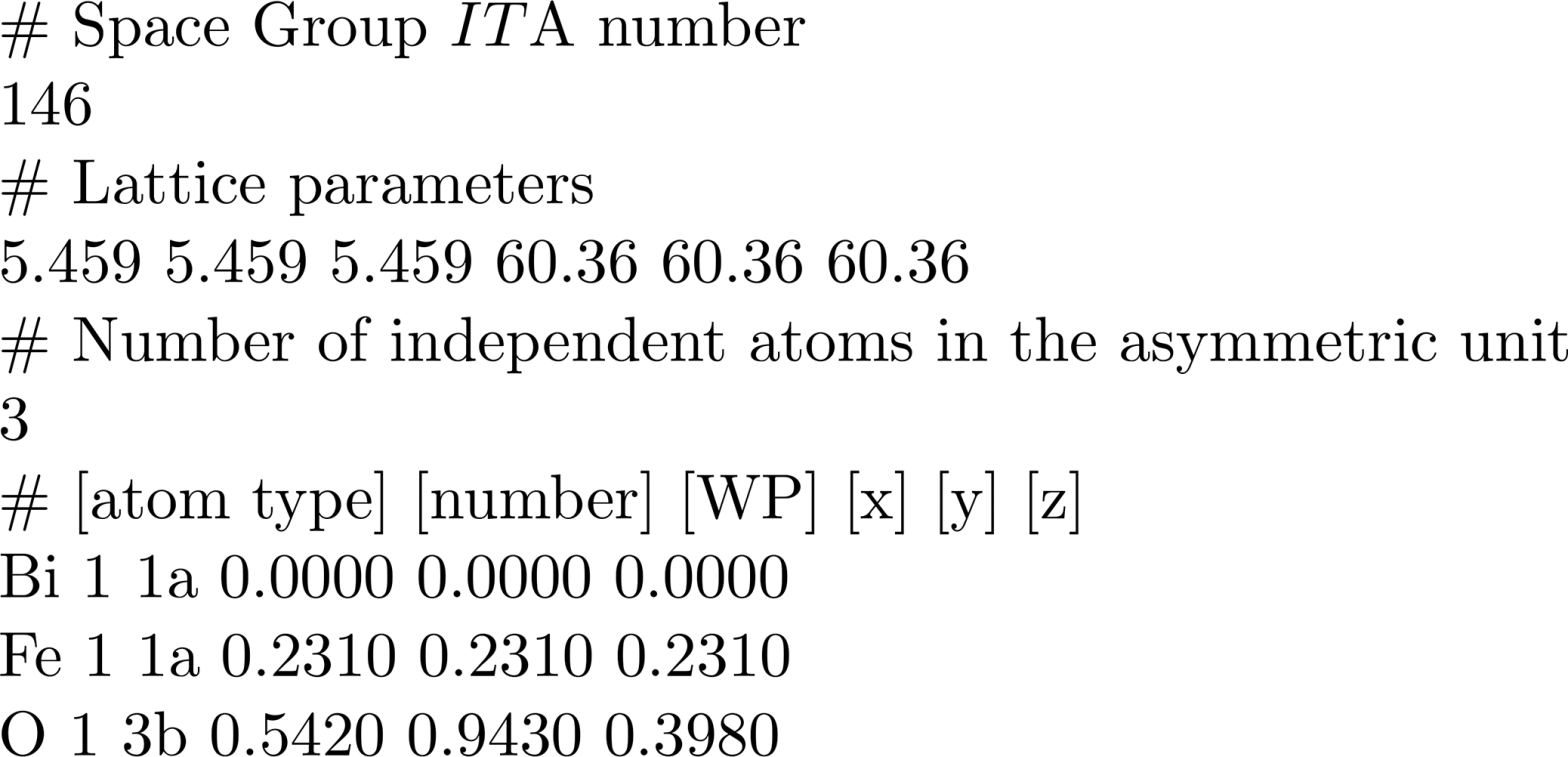


The program *SETSTRU* performs coordinate transformations between *IT*A settings, and the program *TRANSTRU* can transform structural data to any space-group setting and can also be used to transform the unit cell and coordinates of a structure to those of a subgroup. [Note that there are other programs such as *VESTA* (see Section 4), *Mercury* (see Section 3.7) and *JANA* (Petříček *et al.*, 2023 ▸) (https://jana.fzu.cz/) that can perform coordinate transformations and transitions into subgroups. Recently, *Jmol* has also been adapted to be able to calculate and visualize group–subgroup relationships.] Practical examples of using the programs *SETSTRU* and *TRANSTRU* are available in the videos ‘*Example SETSTRU*’ and ‘*Example TRANSTRU*’, respectively (files numbered 7 and 8 in the supporting information).

The program *COMPSTRU* (de la Flor *et al.*, 2016 ▸) is used to quantify the similarity of two structural models of the same phase or structures with different compositions which are isopointal, *i.e.* structures belonging to the same space-group type (or space groups that form an enantiomorphic pair), described in their standard settings, with the same or different chemical compositions and having the same sequence of occupied Wyckoff positions. The program *STRUCTURE RELATIONS*, however, is able to find the relation between two crystal structures having the same chemical composition and whose space groups are group–subgroup related. This program is used for the analysis and characterization of crystalline-state phase transitions with no change in the chemical composition. It calculates the index of the symmetry reduction and the transformation matrix–column pair (**P**, **p**) relating the coordinate system of the group to that of the subgroup. More details about the use of the programs *COMPSTRU* and *STRUCTURE RELATIONS* are given in Section 6 and in the videos ‘*Example COMPSTRU*’ and ‘*Example STRUCTURE RELATIONS*’ (files numbered 9 and 5 in the supporting information).

## 3. *Mercury* and the CSD

### 3.1. Introduction to visualization software *Mercury*

Symmetry elements in structures in the CSD (Groom *et al.*, 2016 ▸) or any structure stored in CIF format can be explored using the CCDC visualization program *Mercury* (Macrae *et al.*, 2020 ▸). Both experimentally determined and computationally generated structural models can be read directly into the free version of *Mercury* and symmetry can be visualized. *Mercury* runs on Microsoft Windows, macOS and Linux and the supported platforms are kept up to date on an FAQ (https://www.ccdc.cam.ac.uk/support-and-resources/support/case/?caseid=f8eb6b5e-0e54-4867-8b01-b21e84299e81). The installers for each of these three platforms can be downloaded from the CCDC website after signing in or registering for a free CCDC account. Once downloaded, the file needs to be double clicked and the instructions in the installer dialogue followed. Once installed, free *Mercury* will need to be activated by using the *CCDC Activator* tool and selecting the *CSD-Community* option.

The *Mercury* software is available to download for free, with more advanced features available with a CSD licence (https://www.ccdc.cam.ac.uk/discover/blog/the-difference-between-free-mercury-and-full-licence-mercury/). The free version of *Mercury* is designed to allow the exploration and visualization of 3D structures and enables users to generate high-quality graphics and movies for scientific communication. Functionality free *Mercury* includes a comprehensive range of visualization features, the ability to explore crystallographic structures through their longer-range interactions such as hydrogen bonds, short contacts and polymer expansion, the generation of high-resolution images and files to 3D-print molecular and crystallographic structures, and the ability to simulate powder patterns and display the symmetry within a structure. *Mercury* functionality referenced in this section is all contained in the free version of *Mercury*, which has an accompanying instruction manual (https://www.ccdc.cam.ac.uk/support-and-resources/documentation-and-resources/?category=All%20Categories&product=Mercury&type=User%20Guide) and training resources (https://www.ccdc.cam.ac.uk/community/training-and-learning/).

### 3.2. Retrieving symmetry information from the CSD

For small organic, metal–organic and inorganic substances, the joint CCDC and FIZ Karlsruhe Access Structures website (https://www.ccdc.cam.ac.uk/structures/) is a helpful and comprehensive source of published experimental structural data. Access Structures enables users to view and retrieve any individual deposited data set in the CSD or ICSD, which together contain over 1.5 million crystal structures. Each entry in the CSD is assigned a database identifier known as a CSD Refcode (https://registry.identifiers.org/registry/csd) to enable individual data sets and structures of the same substance to be retrieved more easily. More advanced searches of the CSD can be performed using the CCDC desktop software *ConQuest* (Bruno *et al.*, 2002 ▸) and web platform *WebCSD* (https://www.ccdc.cam.ac.uk/solutions/software/webcsd/). In these applications users can click on the space-group number to link to *IT*A to explore the space-group symmetry in more detail.

### 3.3. Educational resources available alongside the CSD

Alongside the CSD, the CCDC collates a collection of structures specifically chosen to help educators demonstrate key chemical concepts and teach others about chemistry and crystallography. This collection is called the CSD Teaching Subset (Battle *et al.*, 2011 ▸) and this curated resource contains over 800 structures classified by the various concepts they can be used to demonstrate. Structures annotated as being in the symmetry category of this subset contain molecules that exhibit the most common point-group symmetries. The CSD Teaching Subset can be accessed in full and visualized in three dimensions in *Mercury* from the *CSD-Community* menu (*CSD-Community* > *Open Teaching Database*). Each individual structure can also be viewed and retrieved from the Access Structures website, with the individual categories linked from the CSD Teaching Subset website (https://www.ccdc.cam.ac.uk/community/education-and-outreach/education/teaching-subset/).

To complement the CSD Teaching Subset there is a collection of teaching modules (https://www.ccdc.cam.ac.uk/community/education-and-outreach/education/teaching-modules/), realized in collaboration with educators in the crystallographic community, which can be used as standalone educational packages for a broad range of chemistry science topics, including symmetry. To aid learning further there is also a collection of educational videos that are designed to teach students about symmetry and these are available through a *Symmetry Operations and Symmetry Elements* playlist (https://www.youtube.com/playlist?list=PLEtBZ08SGIScECmQvgvhZ0qXP3WWlBTnv).

### 3.4. Displaying symmetry in *Mercury*

**Figure 6:**
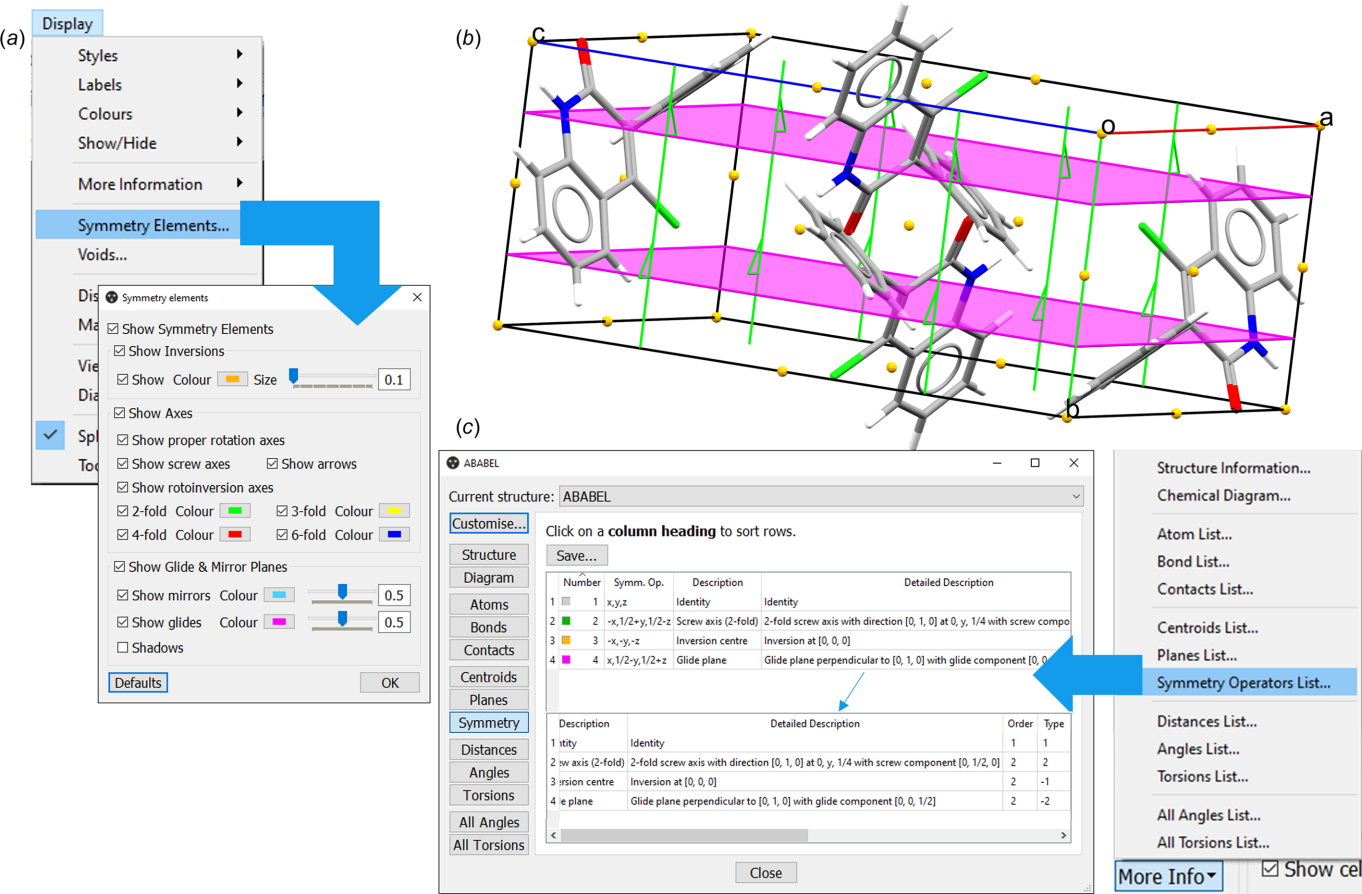
Symmetry information and visualization in *Mercury* for the structure of 4-chloro-3-phenylquinolin-2(1*H*)-one, CSD entry ABABEL (Li *et al.*, 2004 ▸), from the CSD Teaching Subset. (*a*) The *Mercury Display* menu and the *Symmetry Elements* wizard. (*b*) A unit-cell view of CSD entry ABABEL. On the structure are displayed the geometric elements associated with the symmetry operations for it. (*c*) The menu to retrieve the *Symmetry Operator List* in *Mercury*, and a table with such a list, including the information highlighted in the main text. The example shown is for CSD entry ABABEL.

*Mercury* allows the user to visualize the geometric elements of the symmetry operations of a crystal structure in three dimensions, overlaid on the crystal structure (Fig. 6 ▸). This feature can be activated from the *Display* menu by selecting *Symmetry Elements* and, in order to see how these elements are applied in the full structure, crystal packing can be displayed from the *Calculate* menu by selecting *Packing* and *Slicing*. In the *Mercury* software geometric elements are often referred to as symmetry elements in order to differentiate them from functionality used to explore intramolecular geometries and molecular conformations. The elements of the symmetry operations will be displayed on one unit cell; by default, they are represented as follows:

(i) Inversion points are shown as orange spheres.

(ii) Rotation axes are shown as lines and screw axes as lines with half-arrow heads, coloured according to the order of rotation.

(iii) Glide and mirror planes are represented by planes, in magenta for glides and blue for mirrors.

The *Symmetry Elements* wizard interface will allow the user to personalize the display of the elements, as well as toggle them on and off.

It is also possible to learn more about the symmetry operations associated with each element. Clicking on the *More Info* button and selecting *Symmetry Operators List* will bring up a table with the list of symmetry operators, and for each a detailed description and further information, including a colour key matching the default colour of the elements displayed.

**Figure 7:**
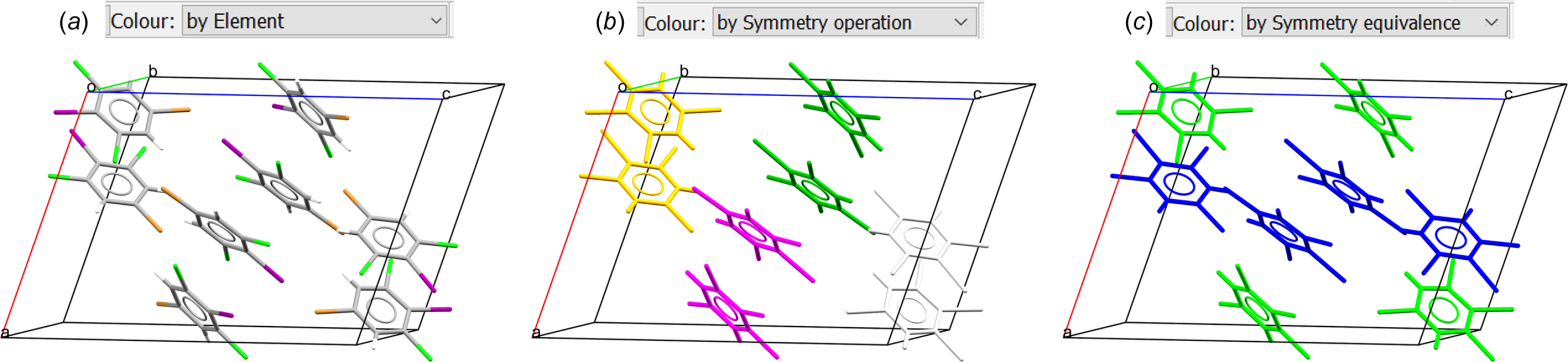
The colouring options in *Mercury* exemplified on CSD Refcode ACEPOO (Arun Prasad *et al.*, 2004 ▸), 5-bromo-1,3-dichloro-2-iodobenzene. (*a*) The atoms are coloured *by Element*, *i.e.* assigning a colour according to their chemical element. (*b*) The atoms are coloured *by Symmetry operation*, *i.e.* according to the symmetry operation that generated them. (*c*) The atoms are coloured *by Symmetry equivalence*, *i.e.* atoms in crystallographically identical molecules are coloured the same.

To complement further the visualization of symmetry in crystal structures, *Mercury* allows the user to colour molecules and atoms according to the symmetry relationship they have to the asymmetric unit (Fig. 7 ▸). This can be done from the *Colour* dropdown menu, by selecting *by Symmetry operation*. An additional colouring option of interest is *Colour: by Symmetry equivalence*. This colour scheme assigns the same colour to different molecules or ions that are crystallographically identical. The video ‘*Symmetry Features in Mercury*’ in the supporting information (file number 3) gives a detailed explanation of how to retrieve symmetry information from the CCDC, and an extended version of this video is also available on YouTube (*How to View Symmetry Elements and Operations in Mercury*, https://www.youtube.com/watch?v=umxCcRFDdss?si=twYSuwOJhYXx73_k).

### 3.5. Additional symmetry features

If the user is interested in exploring the contacts in a structure, more can be learned about it and the atoms involved in each contact pair from the *More Info* button by selecting *Contacts List*. The information displayed in the table will include the symmetry operation associated with both atoms.

It is also possible to visualize a network of symmetry-generated molecules. This works by changing the *Picking Mode* in *Mercury* to *Reveal Symmetry-Generated Molecules* from the drop-down menu. Clicking on any point in the visualizer will bring up the closest symmetry-generated molecule. From the *More Info* button the user can also select *Structure* to see more information about the entry, including the space group. From here the user can click on the space-group number to link to *IT*A. The space group of the structure is also displayed alongside the CSD Refcode in the *Structure Navigator* toolbar.

**Figure 8:**
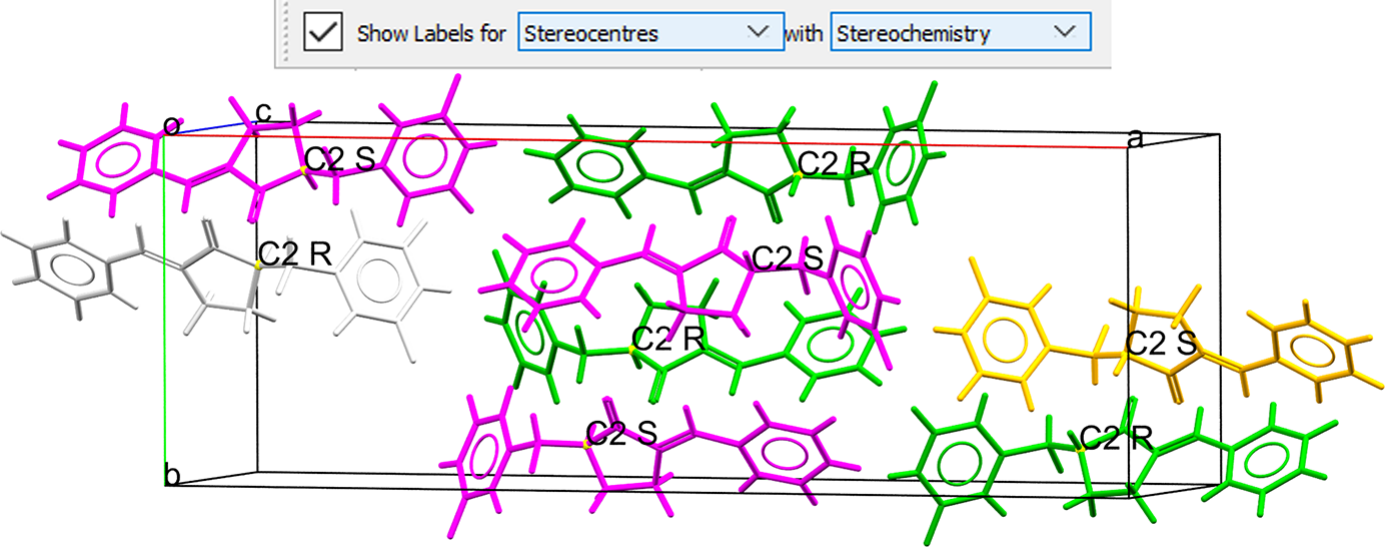
An example of the use of labels to identify stereocentres for CSD Refcode BBZCPO12 (Honda *et al.*, 1999 ▸), 2-benzyl-5-benzylidene­cyclo­pentanone. It can be observed how, for the molecules in this structure, the stereochemistry of the carbon atom C2 changes from *R* to *S* in molecules generated by a glide reflection.

Another feature is the possibility of using *Mercury* to identify stereocentres (Fig. 8 ▸) simply by using labels. *Mercury* allows the user to select the atoms they wish to show labels for, in this case *Stereocentres*, and which atom properties they wish to display in the label, for example *Stereochemistry*. As a result, once the user clicks on *Show Labels*, all stereocentres will be labelled with their atom label (element + number) and associated stereochemistry letter.

### 3.6. High-impact visualization of structural symmetry

**Figure 9:**
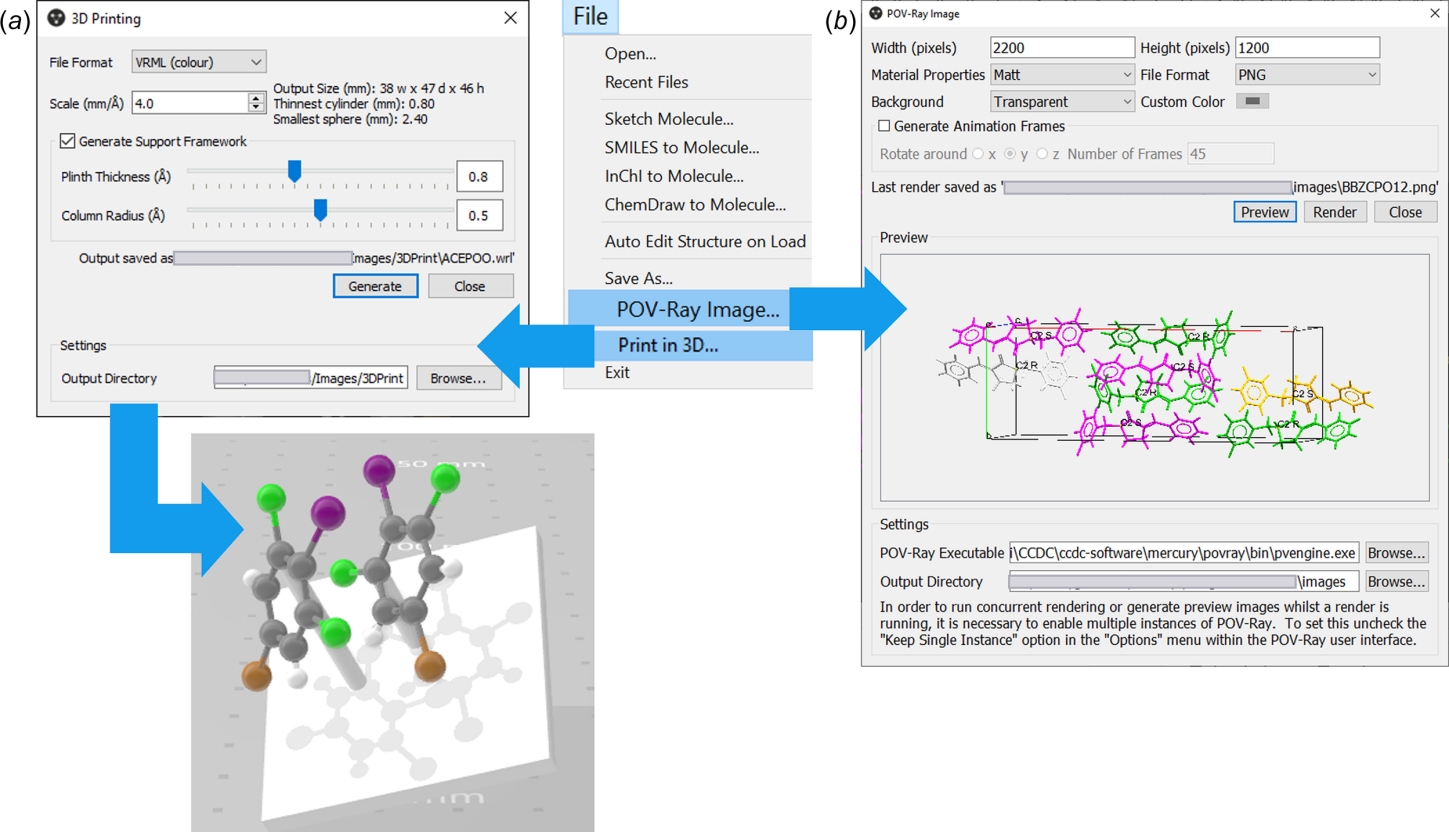
(*a*) The *3D Printing* and (*b*) the *POV-Ray Image* interfaces in *Mercury*, both accessed from the *File* menu. The 3D preview was obtained as a screenshot from opening the file created by *Mercury* using the *3D Printing* program on a machine running Windows.

To communicate research outcomes effectively or to teach symmetry-related concepts, once the user has explored the symmetry in a crystal structure, they may want to create images or animations. In *Mercury* one can generate high-quality publication-ready images using *POV-Ray* (*POV-Ray – The Persistence of Vision Ray Tracer*, https://www.povray.org/) [Fig. 9 ▸(*b*)]. This functionality is accessed from the *File* menu by selecting *POV-Ray Image*. The *POV-Ray Image* interface will allow the user to customize the settings, such as the image size in pixels and the background colour, which includes a transparent option as well. If the user wishes to generate an animation, such as a GIF or a video, showing a structure rotating around an axis, they should click *Generate Animation Frames* and *Mercury* will generate frames for the rotation (as many as are assigned in *Number of Frames*); these frames can then be assembled into a GIF or video, for example, using third-party software.

*Mercury* additionally allows the creation of files for 3D-printing structures or molecules [Fig. 9 ▸(*a*)]. This functionality is also accessed from the *File* menu, by selecting *Print in 3D*. The settings can be personalized from the *3D Printing* interface, including the file format, the scale and whether to include a support framework.

### 3.7. Advanced functionality for structural comparisons

In *Mercury* it is possible to compare and overlay structures and molecules, as well as to change the space-group settings and reduce the symmetry. This functionality can be particularly powerful when comparing two related crystal structures, such as polymorphic structures or structures determined at different temperatures and pressures. In the *Calculate* menu of *Mercury* there is functionality to overlay pairs of structures or overlay molecules, and multiple structures can be displayed and manipulated using the *Multiple Structures* dialogue under the *Structure Navigator*. In the *Edit* menu there is functionality to transform, invert or translate molecules within a structure, change the space-group setting, invert crystal structures, remove lattice centring, reduce the symmetry, transform the structure to a reduced cell and change the space group to a subgroup. This advanced functionality is available in the licenced/full version of the software and can be a powerful tool for structure comparisons and analysis.

## 4. Three-dimensional structure visualization with *VESTA*

*VESTA* is a cross-platform program for 3D visualization and investigation of crystal structure data, volumetric (voxel) data and crystal morphology data. It runs on three major operating systems, Microsoft Windows (Version 7 or newer), macOS (Version 10.9 or newer) and Linux. For each platform, it is distributed as a compressed archive file (https://jp-minerals.org/vesta/en/download.html). Once it has been uncompressed, the executable file can be directly executed without requiring any additional installation process.

When crystal structure data are loaded, the following basic information on the input data is output to the *Text Area*: space-group information, unit-cell parameters and volume, a series of crystallographic sites and their site multiplicities, Wyckoff letters, and site-symmetry symbols. On selection of atoms and bonds, further information is output to the *Text Area*:

(i) Symmetry operations and translation vectors, by which the selected atoms are generated from the input coordinates.

(ii) Internally used orthogonal coordinates (Cartesian coordinates).

(iii) Principal axes (in both Cartesian and fractional co­ordinate systems) and their mean-square displacements of anisotropic displacement parameters (ADPs) when atoms are rendered as ADP ellipsoids.

(iv) Interatomic distances (*l*_*i*_), bond angles and dihedral angles with their standard uncertainty (s.u.) if those of the unit-cell parameters and fractional coordinates are supplied.

Selecting *Coordination polyhedra* gives the following physical quantities in addition to information on each site and bonds:

(v) Polyhedral volumes.

(vi) Distortion indices (Baur, 1974 ▸).

(vii) Quadratic elongations (Robinson *et al.*, 1971 ▸).

(viii) Bond-angle variances (Robinson *et al.*, 1971 ▸).

(ix) Effective coordination number (Hoppe, 1979 ▸; Hoppe *et al.*, 1989 ▸).

(x) Charge distribution (Hoppe *et al.*, 1989 ▸; Nespolo *et al.*, 1999 ▸).

(xi) Bond-valence sums (Brown & Altermatt, 1985 ▸).

(xii) Bond lengths expected from bond-valence parameters.

Volumetric data such as electron and nuclear densities, Patterson functions and wavefunctions are displayed as isosurfaces, two-dimensional sections with contours or bird’s-eye views. Among a number of features of *VESTA*, we further describe some examples of advanced usage of crystal structure handling.

### 4.1. Conversion of space-group symmetry

*VESTA* allows us to convert the space group of a crystal structure to its supergroup or subgroup. Before changing space group, it would be safer first to convert a crystal structure to *P*1 (No. 1) by clicking the *Remove symmetry* button in the *Edit Data* dialogue box. All the atomic positions in the unit cell will then be generated as independent sites. Although this process is not always necessary, it eliminates some restrictions on symmetry relations between the original and target crystal structures, making it easier to convert a crystal structure properly. When the original and target space groups have different choices of origin, or the conversion requires changes to the principal axes, a user-defined transformation matrix (**P**, **p**) (Section 2.1.1) must be input. The user then presses the *Remove symmetry* button again (it will reset the transformation matrix to the identity matrix) and finally sets the target space group. When the space group is changed to a higher-symmetry group, or when the unit cell is transformed to a smaller one, the same atomic position may result from two or more sites, or atomic positions closely contacting each other around a special position may be generated. In such a case, the redundant atoms can be removed by clicking the *Remove duplicate atoms…* button. Atoms of the same element name closer than a threshold value, which is specified by the user, are merged onto a single site at their averaged position. The example in Section 6.3 shows how to use this feature of *VESTA* in a practical example.

### 4.2. Creation of superstructures and average structures

**Figure 10:**
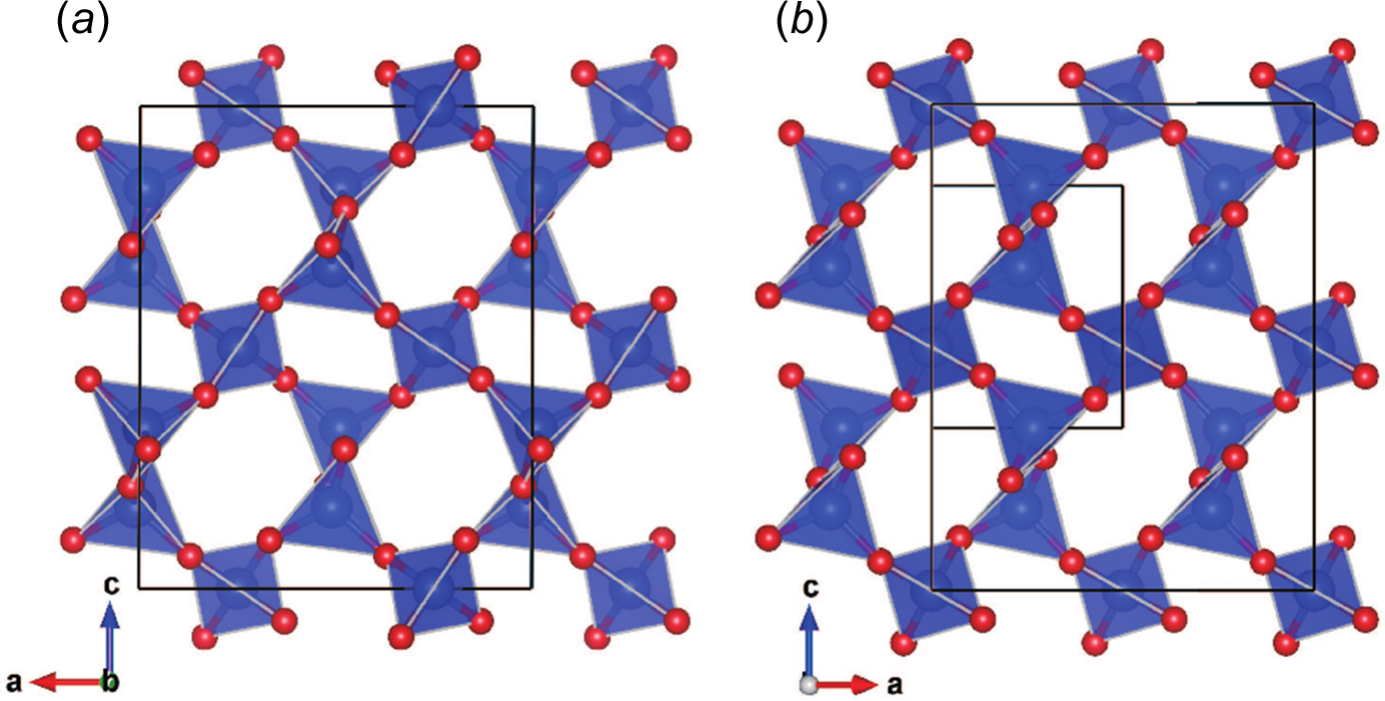
A comparison between two silica polymorphs, (*a*) moganite (*I*2/*a*, No. 15) and (*b*) right-handed α-quartz (*P*3_2_21, No. 154) with its unit cell converted to a monoclinic *C2* (No. 5) space group. The black solid lines show unit cells, and the small rectangle in panel (*b*) shows the original unit cell of α-quartz.

The user-defined transformation matrix is used to convert a standard space-group setting to a non-standard one. The matrix can also be used for transformations between primitive and centred lattices, and for the creation of superstructures and average structures. The matrix transforms both the atomic positions and general equivalent positions (symmetry operations). If the determinant of the matrix is negative, the co­ordinate system is transformed from right- to left-handed, and *vice versa*. When the determinant is larger than 1, the unit-cell volume becomes larger than the original one. In this case *VESTA* can automatically locate additional atoms in the superstructure, either as distinct sites or as additional equivalent positions. When the determinant is smaller than 1, the unit-cell volume becomes smaller than the original one. By clicking the *Remove duplicate atoms…* button, average structures can then be generated from superstructures. Fig. 10 ▸ shows an example of a comparison between two silica polymorphs, moganite (*I*2/*a*, No. 15) and the right-handed α-quartz (*P*3_2_21, No. 154) with its unit cell converted to a monoclinic *C2* (No. 5) space group by a transformation of (**P**, **p**) = (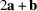, 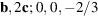).

In addition to transformation of symmetry operations by the transformation matrix (**P**, **p**), symmetry operations may be manually edited. In that case, the symmetry operations do not necessarily form a closed group.

### 4.3. Comparison of multiple structures

*VESTA* allows the user to visualize and compare two or more crystal structures in the same 3D space. This feature facilitates the visualization and analysis of, for example, (i) small differences between similar crystal structures, (ii) interactions between crystal surfaces and non-crystalline molecules or clusters, and (iii) interface structures between two crystals, twins or layer structures with stacking faults between the two.

**Figure 11:**
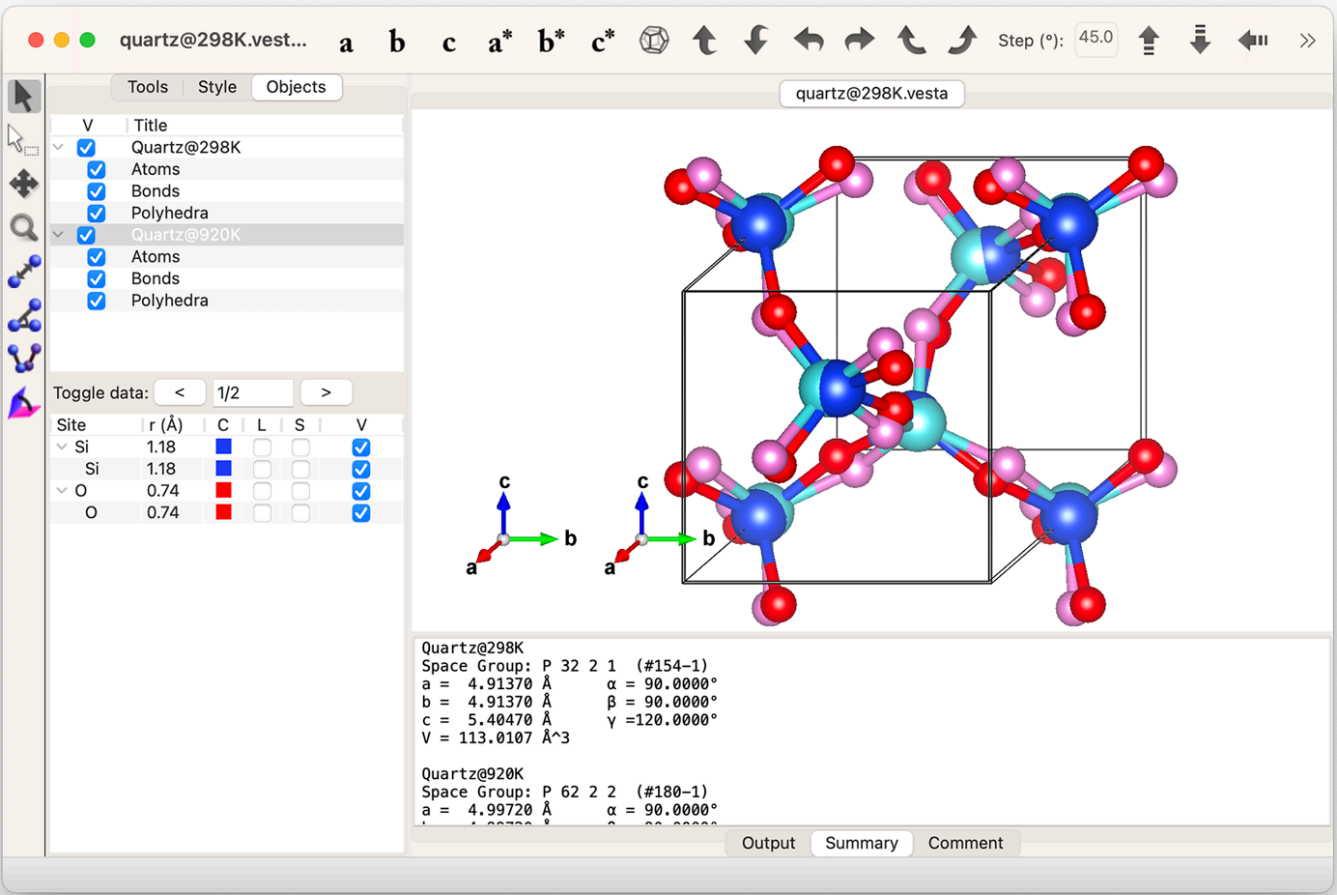
A comparison of the low- and high-temperature forms of right-handed quartz [space groups *P*3_2_21 (No. 154) and *P*6_2_22 (No. 180)] superimposed in a single window of *VESTA*.

**Figure 12:**
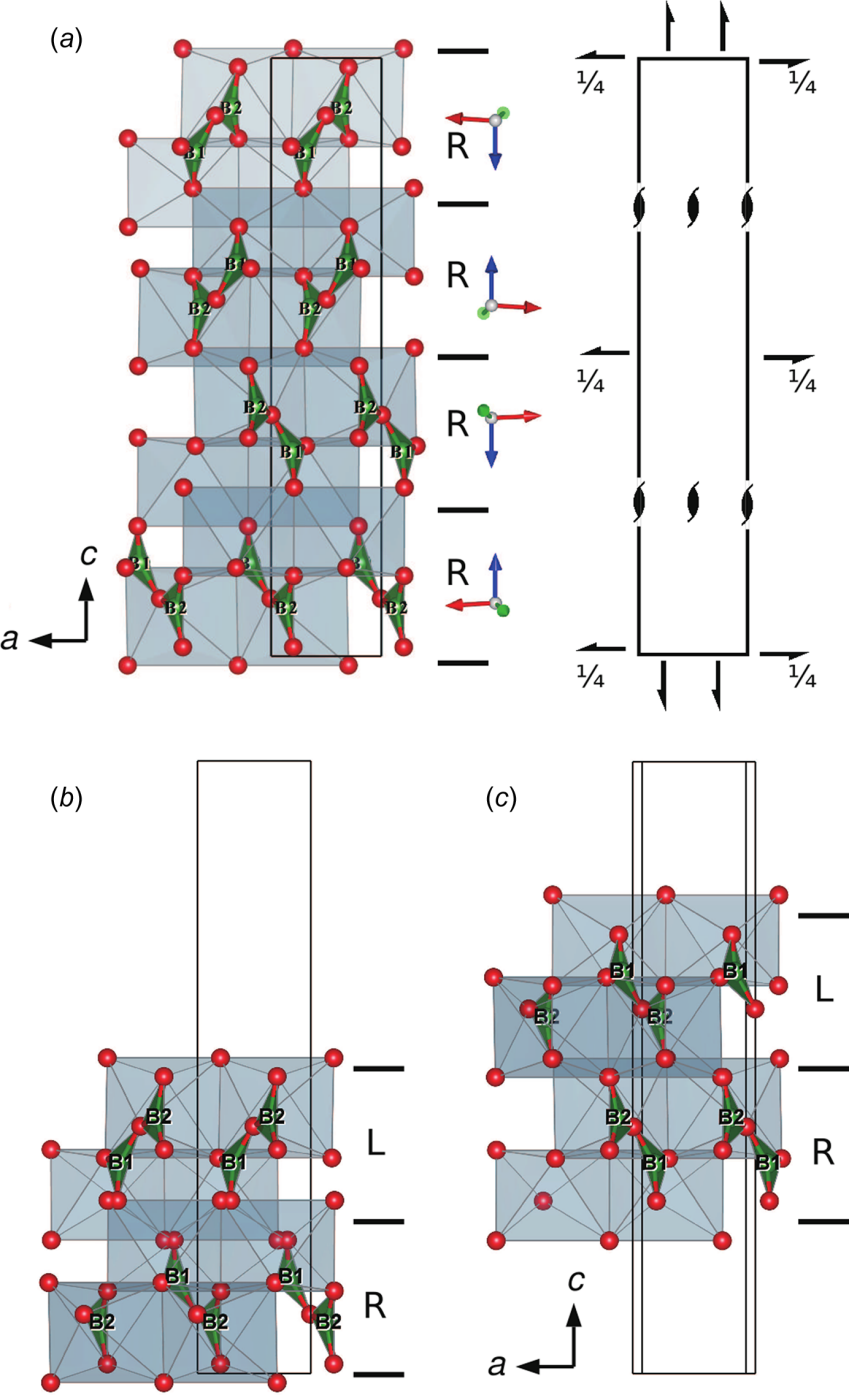
Crystal structures of (*a*) shimazakiite-4*O*, Ca_2_B_2_O_5_, and (*b*) and (*c*) two possible stacking sequences with little or no strain, all drawn by *VESTA*. The 4*O* polytype is built from only one type of layer (enantiomorph) stacking with different orientations. The orientational relation of each layer and the symmetry elements are shown on the right-hand side of panel (*a*). Stacking of the same and enantiomorphic layers (indicated as R and L) gives different monoclinic structures. In the stacking sequence of panel (*b*), a small deformation of the layer is necessary because the oxygen positions of adjacent layers do not exactly match. In the stacking sequence of panel (*c*), no deformation of the layers is required because the oxygen positions match exactly at the interface of the two layers. The actual 4*M* structure is based on stacking sequence (*c*).

Data for second and subsequent phases are input from the *Phase* tab in the *Edit Data* dialogue box. Data can be entered manually, imported from existing files or copied from previously entered data. For each set of phase data, the position and orientation are then set as relative to another phase or the internal Cartesian coordinate system. Fig. 11 ▸ shows an example of a comparison between α- and β-quartz, the low- and high-temperature polymorphs of SiO_2_. The origin of the right-handed α-quartz, *P*3_2_21 (No. 154), is shifted to (0, 0, 2/3) to adjust its position to the standard setting of the right-handed β-quartz, *P*6_2_22 (No. 180). Fig. 12 ▸ shows another example of a comparison of multiple structures actually used to solve a real problem. In this example, the crystal structure of the ortho­rhombic polytype of Ca_2_B_2_O_5_, shimazakiite-4*O* (*P*2_1_2_1_2_1_, No. 19), was solved first, but a monoclinic polytype was also observed (Kusachi *et al.*, 2013 ▸). However, the mono­clinic structure was difficult to solve because of polysynthetic twinning. Starting from the layer structure of the 4*O* polytype, possible stacking structures of the 4*M* polytype were considered using *VESTA*. As a result, one type of stacking sequence was found to be most probable because the oxygen positions exactly matched at the interface of the adjacent layers. A monoclinic structure with space group *P*2_1_/*c* (No. 14) was derived by this type of stacking sequence, and the same structure was later confirmed experimentally by single-crystal X-ray diffraction experiments.

## 5. Web-based visualization of crystal structure and symmetry using *Jmol*

### 5.1. *Jmol* and *JSmol*

*Jmol* (project repositories SourceForge, https://sourceforge.net/projects/jmol, and GitHub, https://github.com/BobHanson/Jmol-SwingJS) is a versatile tool that consists of a stand-alone Java program which, when compiled, is simultaneously produced as an equivalent JavaScript-based web application (sometimes referred to as *JSmol*) using a unique technology (https://github.com/BobHanson/java2script). *Jmol* runs in Java on Windows, MacOS and Linux, as well as in JavaScript in all standard browsers. The Java program is available either with 32-bit floating precision (Jmol.jar) or 64-bit double precision (JmolD.jar). (JavaScript is inherently double precision.) In both contexts, *Jmol* offers two modes: a ‘headless’ command-line mode and an interactive graphical user interface (GUI) mode. Either mode, in Java or JavaScript, can run independently or as a library component of another headless or GUI-based program. As a stand-alone Java program, the headless mode (JmolData.jar or JmolDataD.jar) allows for fully scripted model construction, structure and symmetry calculation, and image production driven by Python or operating system batch scripts.

As JavaScript, *JSmol* can be integrated into web pages in one of three ways. In a headless ‘library’ mode, *JSmol* can support other JavaScript functionality behind the scenes but never be displayed itself. Alternatively, it can be rendered in the form of an independent frame that ‘floats’ on the web page and exactly duplicates its Java implementation. Finally, and most commonly, *JSmol* can be embedded directly into a web application or standard web page surrounded by text and JavaScript components that can drive it and ‘listen’ to it interactively.

In particular, when *JSmol* is embedded within a web page as an interactive component, it becomes part of a larger context. It becomes a window through which the molecular and crystallographic world can be visualized and explored in ways that are customized to that context. For example, we might find *JSmol* being used in a biochemical context to explore protein secondary structure, validation issues or binding sites. We might find it involved in the description of atomic or molecular orbitals in small molecules or periodic systems. It is quite possible, in fact, that a ‘user’ of *Jmol* may not even know that they are using it. All they know is that they are at a dynamic interactive website that lets them do interesting things with atoms and surfaces. The structures involved may be from a database, a study or a publication, or they might be ones the user has ‘dropped’ into the website’s page, allowing the user to explore their own structure within the designed context.

In all cases, whether 32-bit or 64-bit precision, Java, JavaScript, headless, framed or embedded, *Jmol*’s scriptability is its common element. Scripting involves the giving of commands that effect changes in the visualization, that query the application for information about the models, that respond to the way the user is interacting with the models, and that initiate file reading or writing. It is this scriptability that we focus on in this discussion.

The scripting capability of *Jmol* is extensive. The *Jmol*/*JSmol* Interactive Scripting Documentation webpage (https://chemapps.stolaf.edu/jmol/docs) lists approximately 80 script commands, 100 script programming functions, 200 properties and over 400 command parameters. In this section, various snippets of *Jmol* scripting will be highlighted as a way of introducing the reader to some of this functionality. What we will describe here is only a small representative fraction of that capability, with a focus on symmetry, crystal structure visual­ization and crystal structure building. The goal is to give the reader an idea of what is generally available in Java or JavaScript.

### 5.2. Reading crystallographic data into *Jmol*

*Jmol* supports the reading of CIF files of various flavours, including traditional CIF, CIF2, magnetic CIF, modulated structures CIF (both magnetic and nonmagnetic) and topological CIF (all defined in the CIF dictionaries at https://www.iucr.org/resources/cif/dictionaries), mmCIF (*Protein Data Bank in Europe mMCIF Information*, https://www.ebi.ac.uk/pdbe/docs/documentation/mmcif.html), and BinaryCIF (Sehnal *et al.*, 2020 ▸). In addition to reading CIF files, *Jmol* can read over 20 additional experimental and computational crystallo­graphic formats. Additional readers can be added with minimal effort. *Jmol*’s file format ‘resolver’ allows loading files (including drag-dropping into any *JSmol* web application or into the *Jmol* Java application) without any filename-specific requirements.

**Figure 13:**
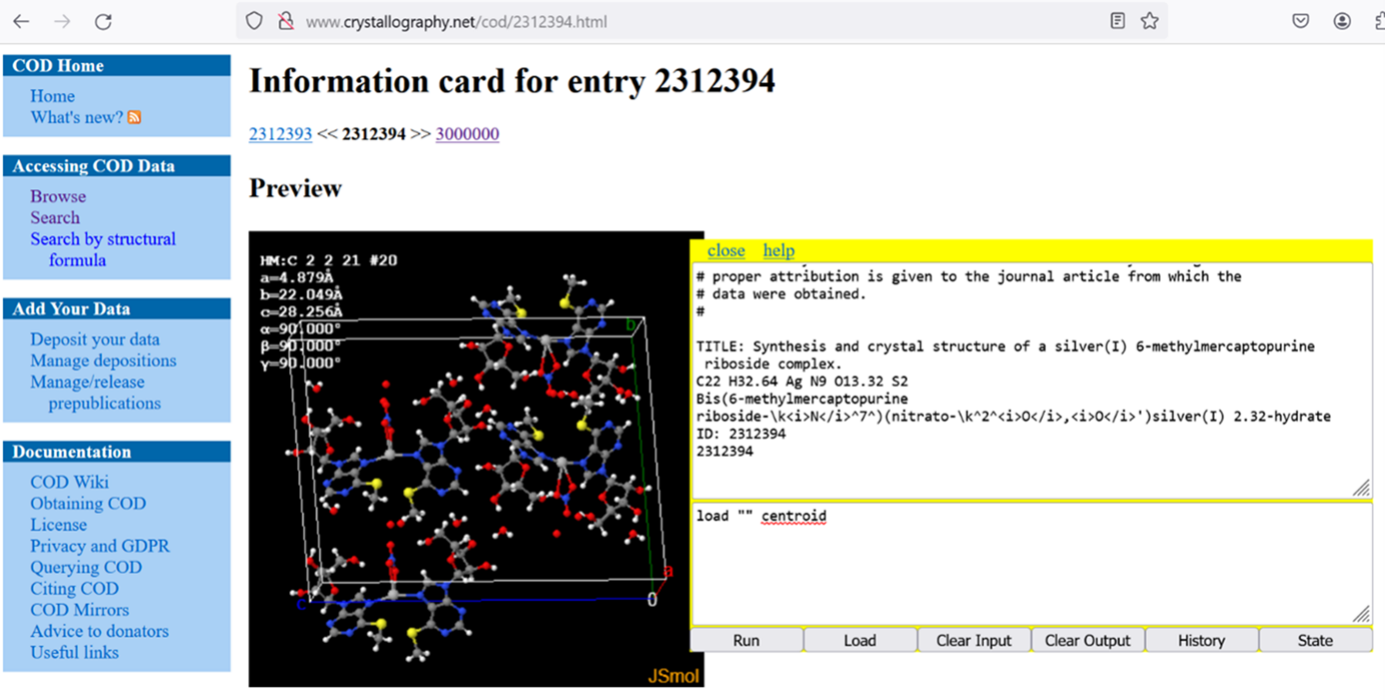
A preview of structure 2312394 from the COD. The (savvy!) user has ‘right-clicked’ the window, pulled up the standard *Jmol* context menu, opened a scripting console and used it to reload the structure with the centroid option (load "" centroid), which completes and displays all molecules with their centre of geometry lying within the representative unit cell.

As for sourcing specific structural data, *Jmol*’s load command includes a number of shortcuts relevant to this discussion. These direct *Jmol* to specific repositories of interest to users. Two of these, PubChem (https://pubchem.ncbi.nlm.nih.gov) and the National Cancer Institute Chemical Identifier Resolver (https://cactus.nci.nih.gov), allow quick loading of small molecules that can be viewed on their own or incorporated into crystal structure models (see Section 6.1). Other databases are focused more on crystallography – most notably the Crystallographic Open Database (load =COD/ id), the American Mineralogist Society Crystal Structure Database (load =AMS/<mineral name>) and a recent addition, the AFLOW Encyclopaedia of Crystallographic Prototypes (load =AFLOWLIB/nnn.m). These three databases represent three very different resource types. The COD contains over 500 000 curated structures deposited primarily by experimentalists (some structures are computational). If a COD structure ID is known, it can be used to load the structure directly into *Jmol*, which is also used on the COD website for previews. In Fig. 13 ▸, we see a *Jmol* model on the COD website, retrievable in *Jmol* using load =cod/2312394. Note that in this example, because of *Jmol*’s inherent scriptability, a website user is not necessarily limited to what the website itself was designed to deliver. In this case, the user has enhanced the model by reloading with the centroid option in order to see better the packing of full molecules in the crystal structure.

Structures from the AMS database also can be accessed by ID, but more interesting is that a mineral name such as *quartz* or *hematite* or *halite* can be used, and in that case the database delivers a collection of structures. Over 1000 minerals are represented, many with several structures, for a total of nearly 6000 structures. For example, load =ams/quartz retrieves 45 models published between 1926 and 2008, with the following distribution, obtained using the *Jmol* script load =ams/quartz; print getProperty("fileInfo.models").select("(_journal_year)").pivot.format("JSON");: {"1926": 1, "1935": 1, "1939": 1, "1962": 2, "1980": 6, "1988": 1, "1989": 6, "1990": 17, "1992": 4, "2000": 1, "2005": 1, "2007": 3, "2008": 1}.

**Figure 14:**
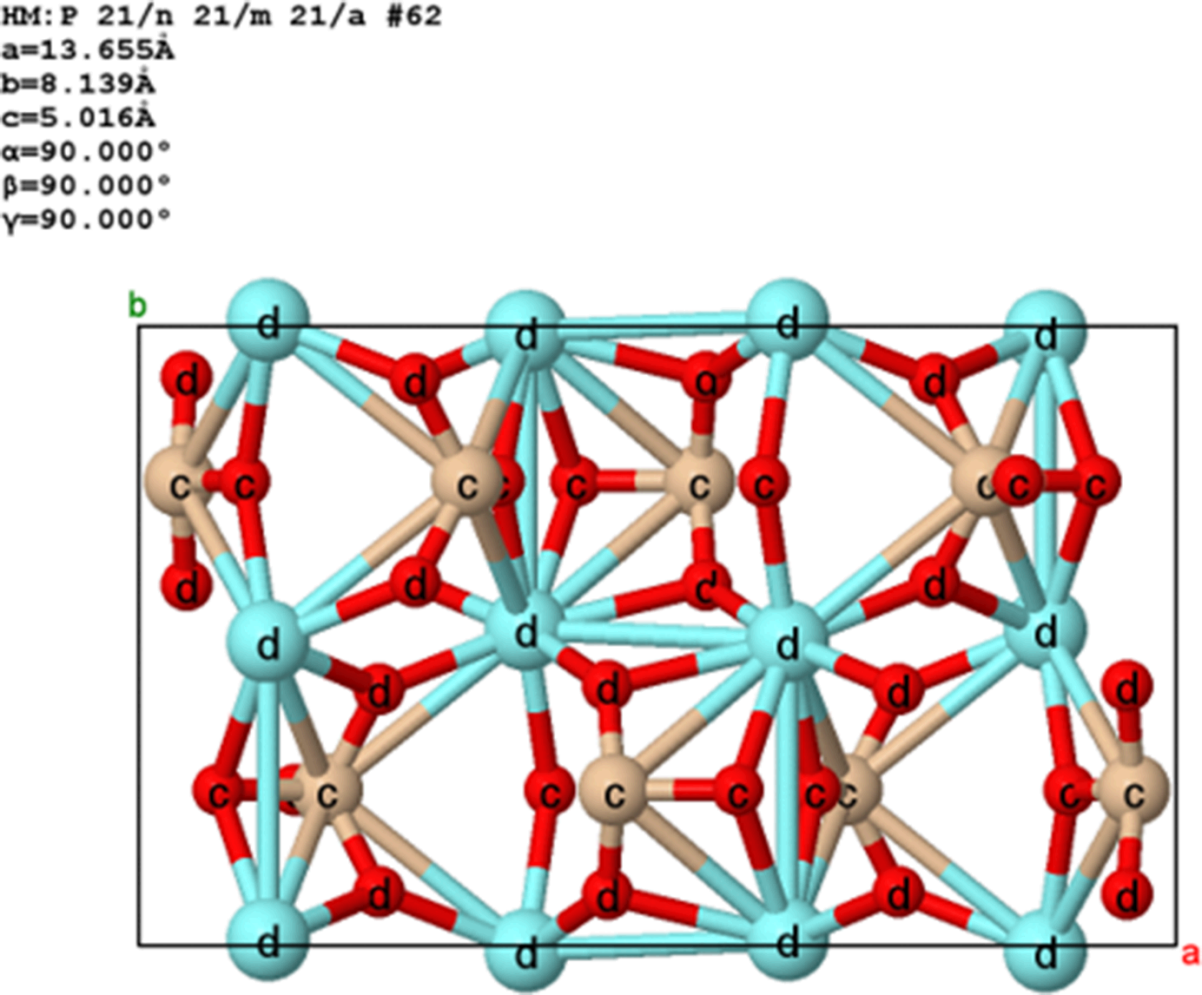
A structure loaded using load =aflowlib/62.77 packed. The atoms have been labelled by their Wyckoff position using select all; label %[Wyckoff]; set labeloffset 0 0;. Additional commands included background white; font labels 20; color labels black;. The view was saved as an ‘enhanced’ PNG image (allowing the model to be shared by email and dragged back into a *Jmol* application or *JSmol* app) using write figure17.png as PNGJ.

In contrast, the AFLOW Encyclopaedia of Crystallographic Prototypes allows for loading one or more example structures from every space group. This database is perfect for use in a teaching or personal exploratory context, where we just want an example of one or more structures for a specific space group. Every space group is represented by from one to 95 structures (*Pnma*, No. 62). An example is shown in Fig. 14 ▸ from the *Jmol* application.

Some of the more powerful features of *Jmol* involve how it loads files. *Jmol* can load crystallographic files with a number of options, all of which are scriptable. There are far too many possibilities to discuss here. Some of the more interesting options, though, are described in Table 1 ▸. Additional options allow the overriding of space group, unit cell and/or origin for the data in the file as a way of investigating differences in symmetry of related subgroups and supergroups.

### 5.3. The *Jmol* crystallographic model kit

It is also possible to create crystal structures easily from scratch using *Jmol*. This is accomplished using simple scripting involving modelkit and related commands. Table 2 ▸ lists some of the many modelkit command options that can be used to build crystal structures. The basic idea is (i) to select a space group, (ii) to define the unit cell and (iii) to add atoms. Over 600 settings of the 230 crystallographic space groups are available. Unit cells are defined using an array, indicating [*a b c* α β γ], or in relation to the current unit cell using the transformation notation as described in Section 2.1.1, such as 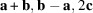.

Atoms can be added to a crystal structure using decimal numbers for Cartesian coordinates in ångströms, such as {1.243, 2.336, 5.731}, or fractional coordinates, indicated by at least one value being expressed as a fraction using a forward slash ‘/’, for example {0 0 1/2} or {0.123, 0.233, 0.500/1}. In addition, atoms can be placed using Wyckoff position labels (*a*–*z* or *A*) or *G* (for the general position). In the case of a Wyckoff position that is not a single point, *Jmol* will place the atom on the Wyckoff position on the basis of an internal default; the user can then move it to a more suitable location within that Wyckoff position if desired. In all cases, *Jmol* will populate all equivalent atom positions. If a specific position is desired, fractional coordinates are recommended, *i.e.* ‘0.333’ is not 1/3.

**Figure 15:**
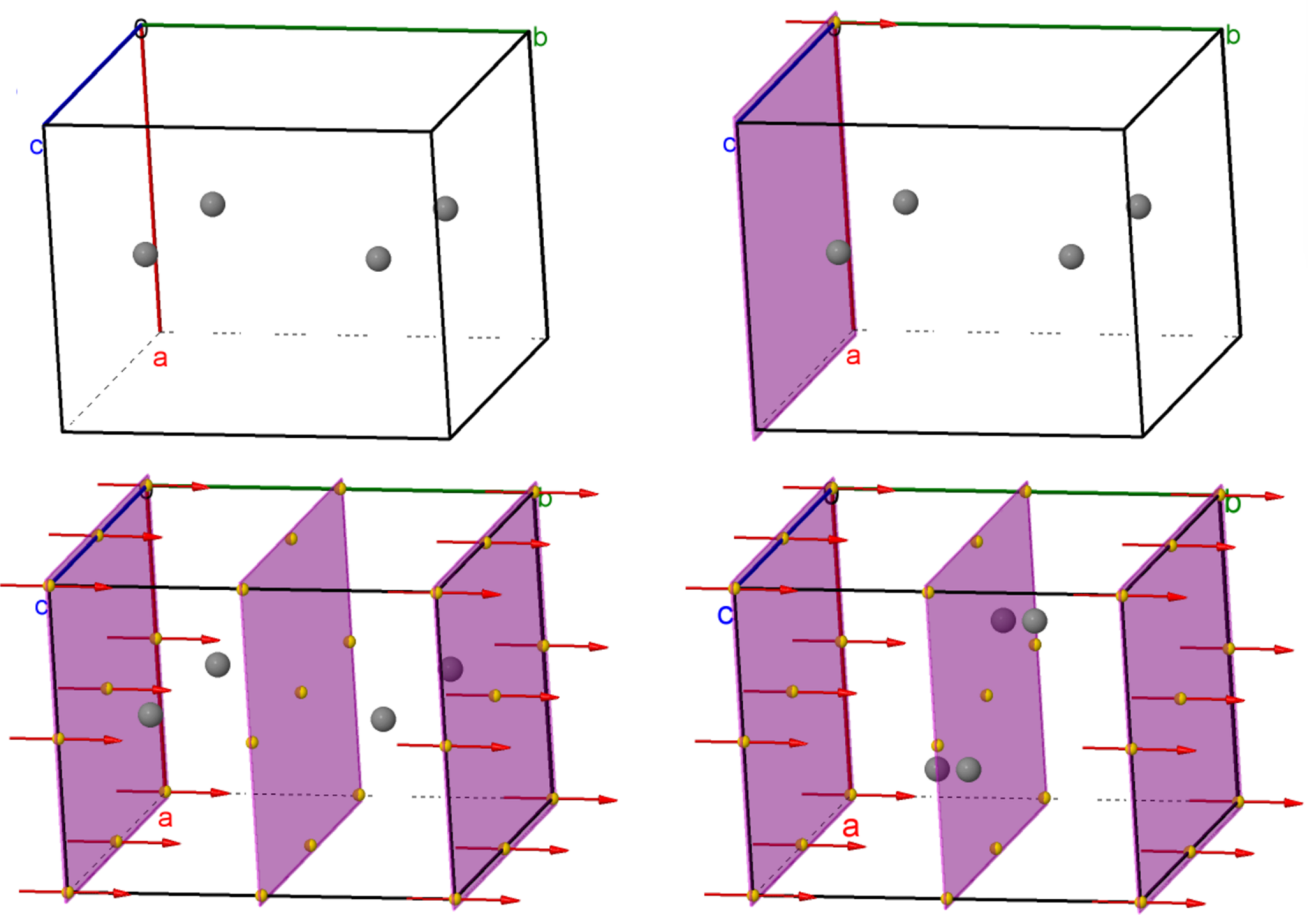
Creating a model using zap; modelkit spacegroup 10; modelkit add C {0.460 0.383 0.288/1}. Adding symmetry elements using draw spacegroup indicates the presence of a mirror plane, a twofold axis of rotation and an inversion centre. Using draw spacegroup all, we see the full set of operations associated with the representative unit cell. After issuing set picking dragatom, an atom was moved with the mouse and the others followed.

So, for example for the model depicted in Fig. 15 ▸, the commands zap; modelkit spacegroup "P2/m"; modelkit add C wyckoff G packed created a model of space group *P*2/*m* (No. 10) which was rotated a bit by hand. Four atoms were added on the general position 4*o*, (*x*, *y*, *z*) (−*x*, *y*, −*z*) (−*x*, −*y*, −*z*) (*x*, *y*, *z*). The command draw spacegroup was used to visualize the twofold axis, mirror plane and inversion centre that characterize this space group, and draw spacegroup ALL completed the diagram. Finally, after issuing set picking dragatom, one of the atoms was clicked and dragged, resulting in all four atoms moving to new symmetry-equivalent positions.

**Figure 16:**
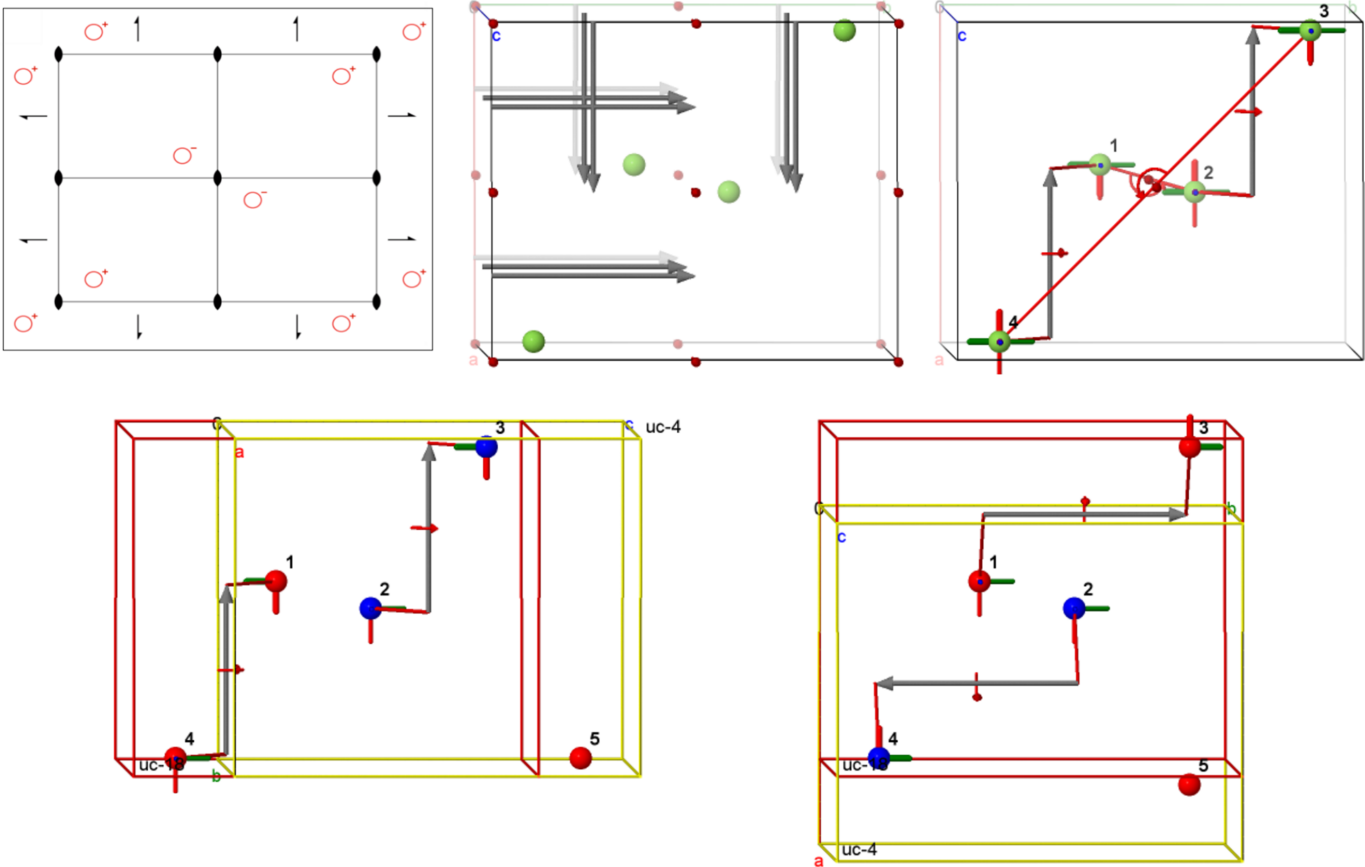
(Top row) A comparison of *P*2_1_2_1_2 (No. 18) and two settings of its maximal subgroup *P*2_1_ (No. 4). One set of equivalent general positions (Wyckoff position 4*c*) was generated using the command modelkit add "F" {0.460 0.383 0.288/1}. The view in all cases is down the *c* axis, looking towards the origin; **a** is downwards and **b** is to the right. The half-unit-long grey arrows are twofold screw axes; red arrows are twofold rotation axes. A Wyckoff orbit is shown using draw symop @1 @2; draw symop @2 @3; draw symop @3 @4; draw symop @4 @1. Multicoloured orientation frames on atoms indicate the orientation change due to rotation. (Bottom row) The same crystal structure, but now in the bases of two different settings of subgroup *P*2_1_. In each case, the red unit cell is the original unit cell and the yellow unit cell is for the subgroup. Atoms are coloured by site, showing the splitting of the general position. The loss of symmetry in each case is apparent by the missing symmetry depictions.

A relatively new capability of the *Jmol Crystallographic Model Kit* (*Jmol* Version 16.2) is the ability to calculate and depict the relationships between super- and subgroups. The top row of Fig. 16 ▸ illustrates space group *P*2_1_2_1_2 (No. 18) as a traditional general position diagram, as a 3D interactive model and as a 3D model showing various symmetry operations relating pairs of general position coordinates. The symmetry in this case involves three generators – two perpendicular twofold screw axes offset from the origin by 1/4 along the *x* axis, and a twofold rotation axis through the origin in the **c** direction. In the second row we see the relationships between the group and two settings of one of its maximal subgroups, *P*2_1_ (No. 4). Using the *Jmol* command color property site, we can see how the four general positions of *P*2_1_2_1_2 are split in each case by the loss of one or the other of the two perpendicular screw axes. The four 3D models were saved as PNGJ files, allowing the images to be dragged back into *Jmol* and investigated or modified further in three dimensions.

### 5.4. *Jmol* mathematical functions

Along with being scriptable, *Jmol* provides an extensive set of scriptable functions that can be used to obtain information about one or more of the currently loaded models, as well as general information about space groups. Two powerful functions are spacegroup() and symop(). The *Jmol*/*JSmol* interactive documentation includes extensive information about these functions. Table 3 ▸ provides some examples of their use to answer common questions about a model. Though in question format, it is to be understood that these queries would be done, at least for *JSmol*, principally by code on a web page as part of an integrated web application.

### 5.5. *Jmol* in the wild

As mentioned already, what sets *Jmol* apart is its versatility. On its own it may seem like just another program, but its true strength lies in its ability to be contextualized to suit any narrative or area of interest. Table 4 ▸ lists just a few of the *JSmol* implementations relating to crystallography and sym­metry that are currently in service on the web. Readers interested in using *Jmol* are encouraged to join the *Jmol* Users List (https://sourceforge.net/p/jmol/mailman/jmol-users), where there are several ‘power users’ who are always willing to assist in the design and implementation of *JSmol* applications and answer even the simplest of questions.

## 6. Application of the four tools for the structural analysis of thiourea at different temperatures and pressures

### 6.1. Symmetry of thiourea molecule

**Figure 17:**
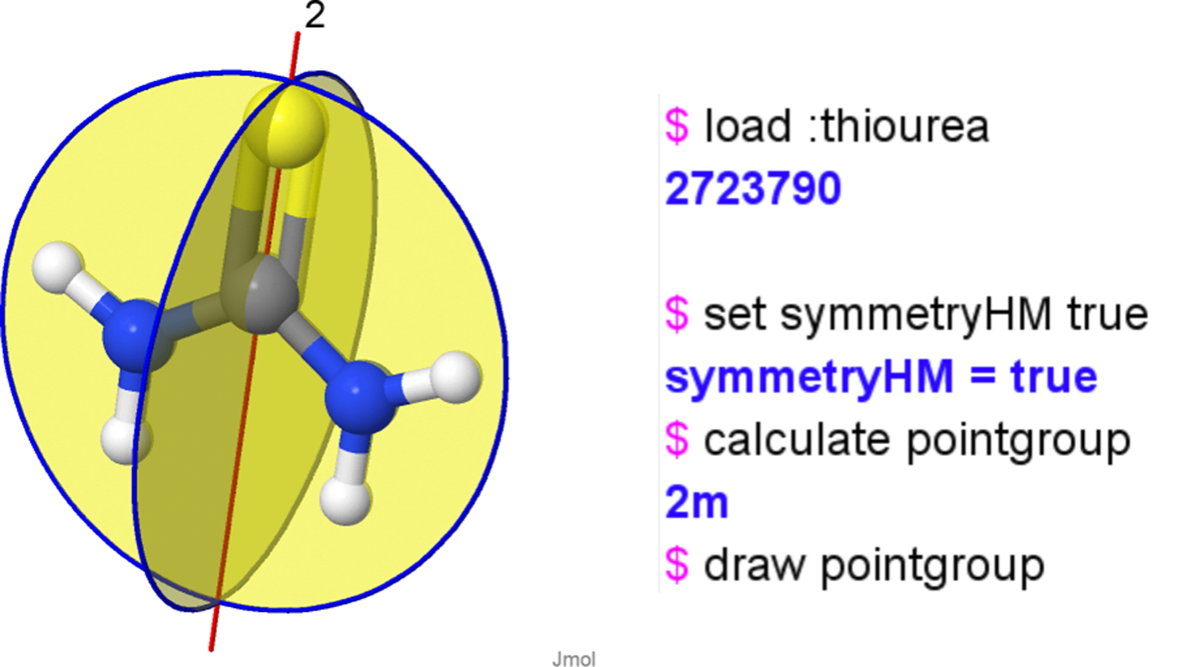
A *JSmol* representation of the thiourea molecule with its twofold axis through the C=S bond, and two perpendicular mirror planes along and perpendicular to the molecule, both containing the twofold axis. Naming of symmetry elements using Hermann–Mauguin symbols is also possible with set symmetryHM.

Thiourea (CH_4_N_2_S) is a synthetic organic compound, an analogue of urea where O is replaced by S.^3^ The molecule is planar and displays *mm*2 symmetry (*C*_2*v*_ in Schönflies notation) where the two NH_2_ groups bonded to C are placed on opposite sides of the C=S molecular axis. In particular, molecules with *mm*2 symmetry can show a permanent dipole moment along the twofold axis, in this case caused by a small negative charge on S and positive charge on H produced by polar C=S and N—H bonds. *Jmol* is capable of determining and visualizing the point group of a molecule, reporting detailed information about the symmetry elements in Hermann–Mauguin or Schönflies notation. In this case thiourea is present in the PubChem database so it can be input into *JSmol* just by opening the console and typing load :thiourea. The symmetry of the molecule can be determined using calculate pointgroup, returning *C*_2*v*_ (or *mm*2). The symmetry operations of the point group can be drawn through the molecule by typing draw pointgroup. The results of these three commands are shown in Fig. 17 ▸.

### 6.2. Crystal structures of thiourea

A search for thiourea in the CSD currently (as of March 2024) yields 33 entries (CSD Refcodes THIOUR and THIOUR01–32), 30 of them including refined atomic co­ordinates (two of them corresponding to deuterated thiourea, CD_4_N_2_S), with the first complete structural refinement made by Truter (1967 ▸) (CSD Refcode THIOUR).^4^ In the group of 28 relevant entries, eight correspond to structure determinations at room pressure and temperature, seven using X-rays and one using neutrons. Ten entries correspond to low-temperature determinations and 11 to high-pressure studies. The video ‘*Downloading cifs from Access Structures*’ in the supporting information (file 4) gives a detailed explanation of how to retrieve the structures of thiourea from the CCDC. The CIF files used in this example are also available in the supporting information (file 1).

**Figure 18:**
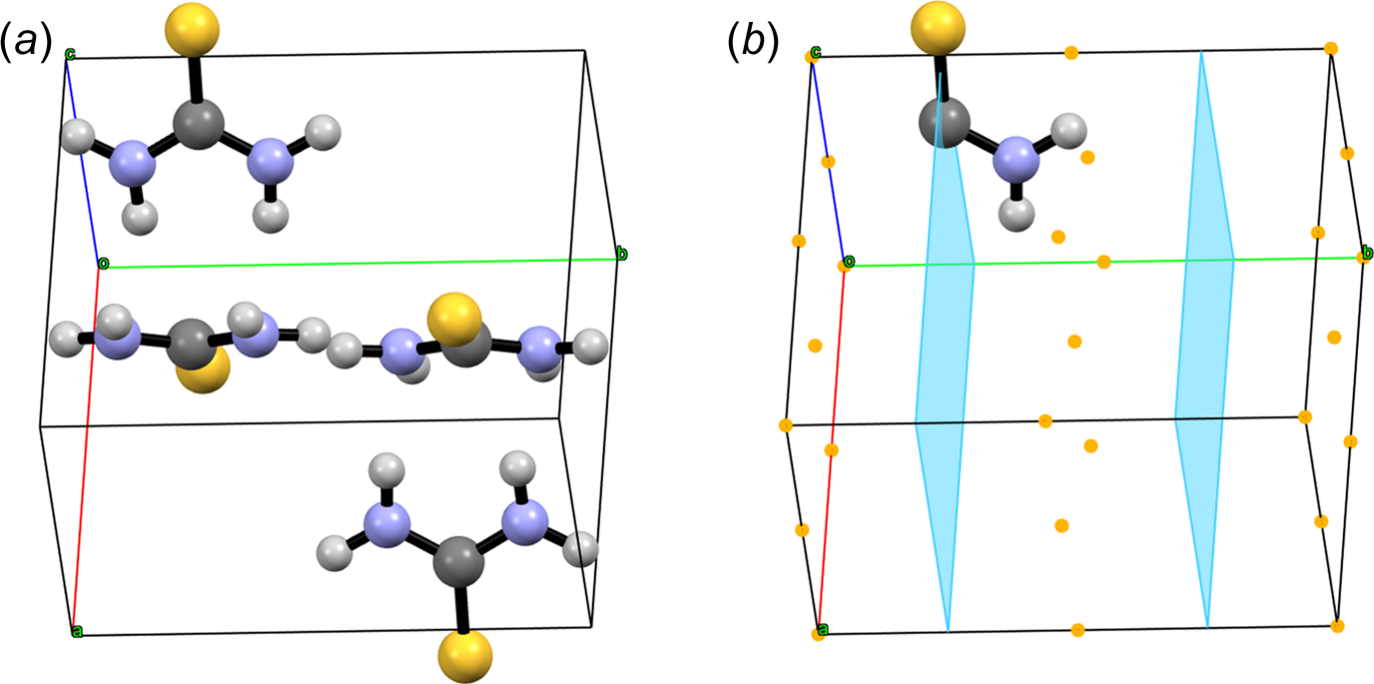
(*a*) The packing of the room-temperature form of thiourea (as given by *Mercury* choosing the *Packing* box) showing four molecules. (*b*) The asymmetric unit of thiourea with symmetry operations containing fixed points (inversion centre and mirror plane parallel to the *ac* plane) of the unit cell (as given by *Mercury* selecting *Asymmetric Unit* and *Auto centre* boxes and *Display/Symmetry Elements* hiding screw axes and glide planes). Note that the contents shown by clicking *Asymmetric Unit* are not necessarily those in the asymmetric unit defined in *IT*A but are in some other visually convenient asymmetric region of the unit cell.

Commercial thiourea can be easily recrystallized from ethanol to yield large colourless rhombic crystals. At room temperature and pressure the crystal structure is ortho­rhombic, space group *Pnma* (No. 62), with *a* = 7.655 (7), *b* = 8.537 (7) and *c* = 5.520 (7) Å, *V* = 360.74 Å^3^ and *V*/*Z* = 90.18 Å^3^, as reported by Elcombe & Taylor (1968 ▸) (CSD Refcode THIOUR01) using single-crystal neutron diffraction data. This crystal structure of polymorph V of thiourea containing four molecules per unit cell (*Z* = 4, *Z*′ = 0.5) is shown in Fig. 18 ▸(*a*). The *mm*2 symmetric thiourea molecule crystallizes with one of its mirror planes coinciding with the crystallographic mirror plane of the space group normal to **b** at *y* = 1/4, making the asymmetric unit of the crystal half a molecule, as shown in Fig. 18 ▸(*b*). This also implies that, in the solid state, the symmetry of the electron density of thiourea is *m*(σ). The difference in symmetry is determined by asymmetric interactions with neighbouring molecules (van der Waals forces in general, hydrogen bonds in this particular case) as discussed by Elcombe & Taylor (1968 ▸).

### 6.3. Comparison of the room- and low-temperature structures of thiourea at ambient pressure

**Figure 19:**
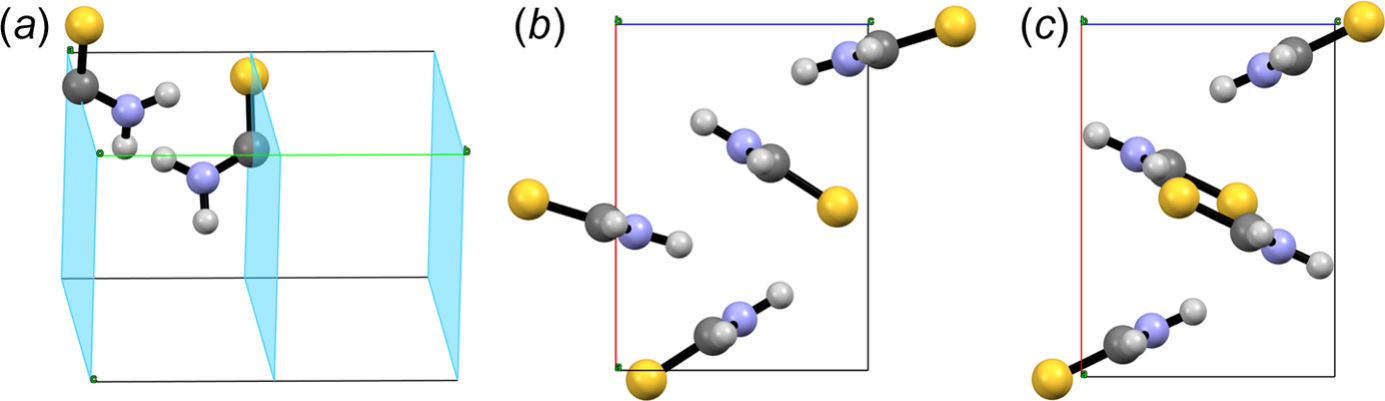
(*a*) The asymmetric unit of thiourea at 123 K, with mirror planes in the *ac* plane of the unit cell. Note that individual molecules keep the same mirror symmetry in both structures, but the loss of inversion symmetry from room to low temperature makes two half-molecules inequivalent in the latter. (*b*) The packing of the *P*2_1_*ma* setting of the low-temperature form of thiourea (as given by *Mercury*). The absence of parallelism between pairs of molecules leaves a net dielectric moment, causing ferroelectricity along the *a* axis. (*c*) The packing of the room-temperature form of thiourea along the *b* axis (as given by *Mercury*), showing the perfectly antiparallel orientation of pairs of molecules preventing ferroelectricity.

Before the first crystal structure determination, it had been reported that room-temperature paralectric crystals of thiourea convert to a ferroelectric phase below 200 K (Solomon, 1956 ▸). One low-temperature form of thiourea called polymorph I that was first reported by Elcombe & Taylor (1968 ▸) is also orthorhombic, space group *P*2_1_*ma*^5^ (No. 26) with *a* = 7.516 (7), *b* = 8.519 (10) and *c* = 5.494 (5) Å, *V* = 351.77 Å^3^ and *V*/*Z* = 87.94 Å^3^ at 123 K (CSD Refcode THIOUR02), maintaining *Z* = 4 but with *Z*′ = 1 [see Fig. 19 ▸(*a*)]. Since the *Pnma* and *P*2_1_*ma* unit cells have very similar cell parameters and share *Z* = 4 but *P*2_1_*ma* is an order 2 subgroup of *Pnma*,^6^ the asymmetric unit of the *P*2_1_*ma* form is twice the volume of the *Pnma* form, leading to a content of two-halves of a thiourea molecule (*Z*′ = 2 × 1/2). In polymorph V, equivalent thiourea molecules are related by an inversion centre, and therefore the electric dipoles that accompany the molecules are antiparallel in the crystal, leaving a zero net dipolar moment. At low temperature inequivalent thiourea molecules are no longer antiparallel and the electric dipoles do not cancel each other, leaving a net polarization along the polar axis (*a* in the *P*2_1_*ma* setting).

The program *STRUCTURE RELATIONS* in the BCS (see Section 2.3) was used to compare the closely related structures of the room- and low-temperature forms of thiourea and quantify the differences. The CIF files obtained from the CSD were used after removing by hand equivalent atoms added by the CSD [atoms N1*G*, H1*G* and H2*G* in THIOUR01.cif and N1*B*, H1*B*, H2*B*, N2*B*, H3*B* and H4*B* in THIOUR02.cif as given in the supporting information (file 1)]. THIOUR01.cif was input as a high-symmetry structure, THIOUR02.cif as a low-symmetry structure, default tolerance values were maintained, and the box informing that one or both of the crystal structures were on a non-standard setting (since *P*2_1_*ma* is an *IT*A setting of *Pmc*2_1_) was selected. On input, the BCS transforms *P*2_1_*ma* into *Pmc*2_1_ and uses the standard setting for the rest of the analysis. After checking the correctness of the BCS listing of both crystal structures, the program determines the transformation matrix needed to represent the high-symmetry structure in the standard setting of the space group of the low-symmetry one. In this case the transformation matrix (**P**, **p**) = (**b**, **c**, **a**; 0.01870, 1/4, 3/4) needs to be applied to go from *Pnma* to *Pmc*2_1_, implying that a change in the names of the axes and a translation of the unit-cell origin are required to make the two structures coincide. Using this transformation, the program calculates all the structural parameters of the first structure into the same setting as the second one, or represents the room-temperature form of thiourea in space group *Pmc*2_1_. This allows an atom-by-atom comparison of position, given in the *Matching Atoms* table, and calculation of the distance between each pair of equivalent atoms, given in the *Differences in Atomic Positions* table, such as the 0.2995 Å displacement of atom S12 relevant to ferroelectric behaviour. Finally, the evaluation of the global distortion of the low-temperature structure with respect to the room-temperature one is calculated in the form of spontaneous strain (*S* = 0.0065), maximum and average atomic displacements (*d*_max_ = 0.3975 Å for H1, *d*_av_ = 0.2497 Å) and the measure of similarity parameter (δ = 0.247 in this case). The video ‘*Example STRUCTURE RELATIONS*’ in the supporting information (file 5) gives a detailed explanation of how to perform these calculations.

Once the transformation of the *Pnma* structure to the *Pmc*2_1_ space group is completed, the new description can be saved to input into the program *COMPSTRU*, which allows a much more detailed mathematical and graphical comparison of the two structures. *COMPSTRU* utilizes *JSmol* to visualize the two models represented in the same settings, highlighting the differences and similarities between the compared structures.

The transformation matrix found by *STRUCTURE RELATIONS* can also be used to convert the *Pnma* model to *P*2_1_*ma* in *VESTA* to transform one model into the other ‘by hand’ (more details about the procedure are given in Section 4.1). THIOUR01.cif is input into *VESTA* and the menu option *Edit/Edit Data/Unit cell* is selected where the *Remove Symmetry* button can be pressed. The *Pnma* model is converted into *P*1. Note that the number of atoms (in the *Structure parameters* tab) changes from five (one C, one S, one N and two H) to 32 (four C, four S, eight N and 16 H). At this point back in the *Unit Cell* tab the *Transform* button can be pressed and a menu will pop up asking for a transformation matrix. The matrix found by *STRUCTURE RELATIONS* can be input to transform the unit cell and coordinates of the *Pnma* model to the most similar *Pmc*2_1_ model. After applying the transformation the unit-cell axes change their name and the positions of thiourea molecules in the unit cell also change. Note that C and S atoms that were located at *y* = 1/4 (the mirror plane **m** || **y** at *x*, 1/4, *z*, the coordinates of the plane where S and C atoms of thiourea are located) in the *Pnma* model are now located at *x* = 0 (the mirror at the *yz* plane) in *Pmc*2_1_. Now the *Pmc*2_1_ symmetry needs to be added. This is done by changing the triclinic space group *P*1 to the ortho­rhombic space group *Pmc*2_1_ (No. 26), setting number 1, *Pmc*2_1_ (**a**, **b**, **c**), but the transformation matrix in the *Transform* window must be initialized first.

After the space-group symmetry has been converted to *Pmc*2_1_, duplicate atoms must be removed in the *Structure Parameters* tab using the *Remove duplicate atoms…* button at the bottom. A threshold of maximum deviation between equivalent atoms can be selected, although the default is fine for exactly matching symmetry-related atoms. Now the atom list changes again from 32 to ten, the new number of atoms in the asymmetric unit of the *Pmc*2_1_ model of thiourea.

This model in *Pmc*2_1_ can be exported as a CIF file and compared with the experimental structure that needs to be transformed in *Pmc*2_1_ for comparison. The *P*2_1_*ma* setting can be obtained simply by selecting setting number 3, *P*2_1_*ma* (**c**, **a**, **b**), and now the unit cell and atomic positions of the atoms become very similar to those in the THIOUR02.cif file. Note they are not identical, since the experimental structure corresponds to an experimental model at 123 K, while the structure obtained by the process just outlined here is a transformation of the room-temperature structure into the same space-group setting.

Note that clicking one atom in a structure in *VESTA* provides the atomic coordinates of that atom and the symmetry operation required to obtain this atom from the one given in the atom list, which is assumed to be the general position *x*, *y*, *z*. Comparing the coordinates of N atoms in the same molecule in the *Pnma* and *P*2_1_*ma* models of the high- and low-temperature forms of thiourea, respectively, allows us to visualize that, in both structures, there is a mirror symmetry relation between the N atoms. But two N atoms in nearby molecules in the asymmetric unit of the cell are not symmetry related in *P*2_1_*ma*. The video ‘*THIOUR01 to 02 transformation VESTA*’ in the supporting information (file 6) gives a detailed explanation of how to perform these calculations.

### 6.4. Comparison of ambient- and high-pressure forms of thiourea at room temperature

**Figure 20:**
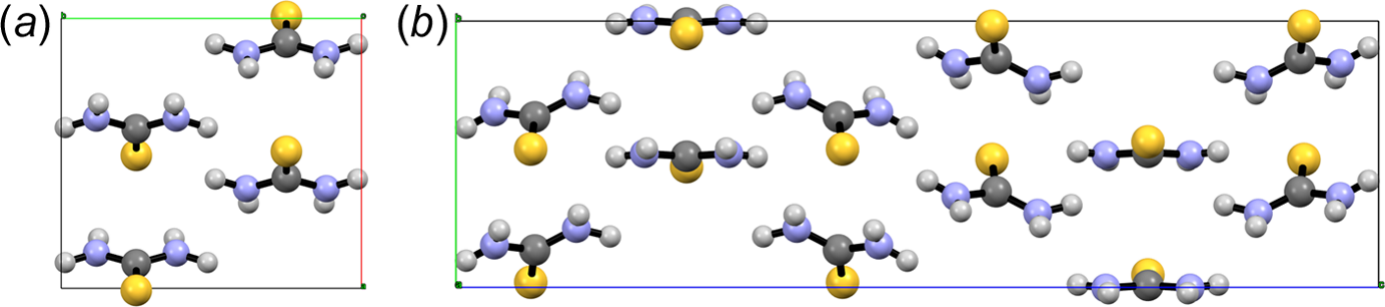
(*a*) The packing of polymorph V (THIOUR01) of thiourea in space group *Pnma* viewed along the *c* axis, and (*b*) the packing of polymorph VI (THIOUR19) of thiourea in space group *Pbnm* viewed along the *a* axis (as given by *Mercury*). In both figures the longest axis (*b* in V and *c* in VI) lies horizontally.

Thiourea crystals have also been studied under applied isostatic pressure. Asahi *et al.* (2000 ▸) determined that, above 0.38 GPa, crystals of polymorph V convert into a new polymorph VI, orthorhombic, *Pbnm* (No. 62), with *a* = 5.503 (3), *b* = 7.138 (4) and *c* = 24.788 (4) Å, *V* = 973.68 Å^3^ and *V*/*Z* = 81.14 Å^3^, as reported at 0.97 GPa (THIOUR19 in the CSD). Note that *Pbnm* is an *IT*A setting of *Pnma* that can be transformed to the standard one using the transformation matrix (**P**, **p**) = (**b**, **c**, **a**; 0, 0, 0).^7^ Fig. 20 ▸ shows the packing of thiourea in polymorphs V and VI side by side. Visual inspection of the unit cells of both forms indicates that the high-pressure form of thiourea shows a tripled cell with respect to the ambient-pressure form. The tripling of the cell is caused by the rotation of two-thirds of the thiourea molecules in the crystal by a small angle along the molecular axis of symmetry, breaking the mirror symmetry of the arrangement and reconstructing the hydrogen-bond network that holds the crystal together. The new space group (of the same type or isomorphic but with a tripled cell) is a subgroup of index 3 of the original one, with only one in every three of the trans­lations along the elongated axis remaining,^8^ and its asymmetric unit is also tripled with respect to the ambient-pressure form containing one and a half thiourea molecules.

In this particular case, visual inspection of the unit-cell parameters and atomic positions allows one to describe the transformation. The same analysis can be performed using *STRUCTURE RELATIONS* and *COMPSTRU* in the BCS. In this case the transformation from the small *Pnma* unit cell to the tripled *Pbnm* one requires the transformation matrix (**P**, **p**) = (**c**, **a**, 3**b**; 0, 0, 0).

**Figure 21:**
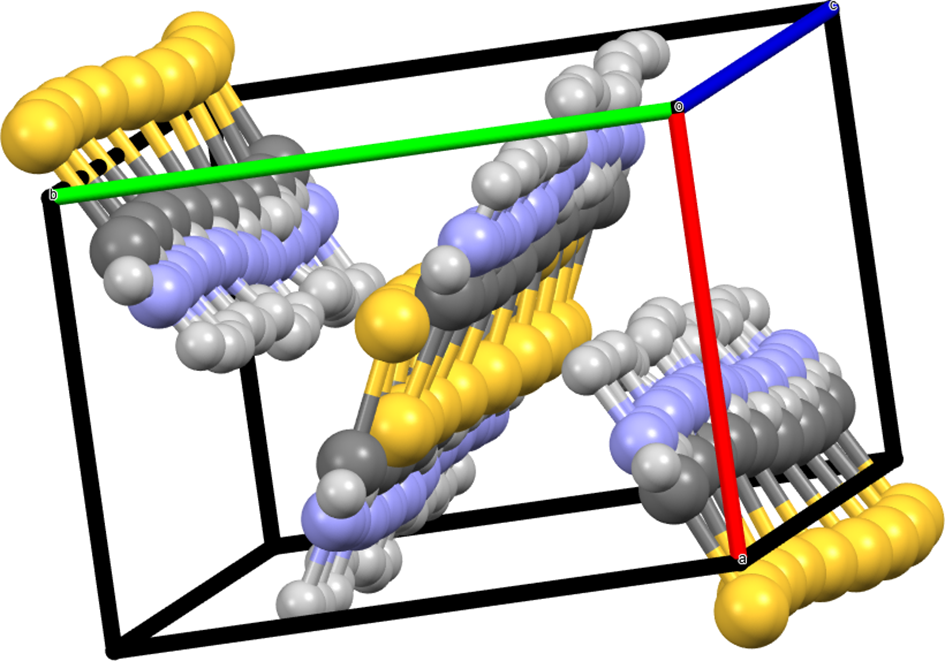
A unit cell of the ninefold superstructure of thiourea called polymorph II′ in entry THIOUR05 of the CSD (as given by *Mercury*), where a sinusoidal positional displacement of molecules is observed close to the commensurate–incommensurate phase transition described by Tanisaki *et al.* (1988 ▸).

The three crystalline forms of thiourea described so far are not the only ones published in the literature. Thiourea forms two incommensurate structures (polymorphs II and IV) on cooling between 160 and 205 K. Only polymorph II has a model included in the CSD (entry THIOUR06). The THIOUR05 entry, referred to as polymporph II′ (see Fig. 21 ▸), contains a periodic phase found in a narrow 2 K range around 179 K and shows a commensurate modulation vector very close to that in polymorph II. The structure of this polymorph II′ was refined by Tanisaki *et al.* (1988 ▸) and found to have an orthorhombic *Pbnm* space group, *a* = 5.467 (1), *b* = 7.545 (1) and *c* = 76.867 (11) Å, *V* = 3170.65 Å^3^ and *V*/*Z* = 88.07 Å^3^. This unit cell corresponds to an order 9 subgroup of the polymorph V structure (note that 8.537 × 9 = 76.833) that contains four thiourea half-molecules per asymmetric unit, and can also be analysed using the tools described above to determine the relation between polymorphs V and VI.

All three graphical tools described in this paper have options to overlap different crystal structures. This can be done by overlapping unit cells with all of their contents (*VESTA*), molecules and packings (*Mercury* in the full version of the program) or average structures (*JSmol* in *COMPSTRU*). These overlap models can help the structure comparison and description of distortions in a visual way that complements mathematical calculations that could quantify them.

## 7. Conclusion

This work collects a set of tools that crystallographers use daily in their research which, among other features, allow the user to analyse, visualize and handle crystallographic symmetry. These resources can also be used for educational purposes, offering valuable tools for teaching crystallography at all educational levels. The characteristics and utilities regarding symmetry analysis of the Bilbao Crystallographic Server, *Mercury* within the Cambridge Structural Database, *VESTA* and *Jmol/JSmol* are described in detail. The application of all the tools described to the comparison of different closely related polymorphic forms of thiourea is also included in the final part of the paper for the reader to reproduce the analyses and use them in the classroom.

The authors hope that this publication, first discussed during a workshop at the IUCr 2023 Congress (Melbourne, Australia), will allow crystallography lecturers and practitioners to feel more confident with non-automated analysis of crystallographic symmetry in their classes or everyday practice, using some of the best tools freely available for the task.

## Supplementary Material

CIF files for the examples in Section 6. DOI: 10.1107/S1600576724007659/dv5017sup1.zip

Web-based visualization of crystal structure and symmetry using Jmol. DOI: 10.1107/S1600576724007659/dv5017sup2.pdf

Video Symmetry features in Mercury. DOI: 10.1107/S1600576724007659/dv5017sup3.mp4

Video Downloading CIFs from Access Structures: related to the example in section 6 that shows how to retrieve the thiourea CIF files from the CCDC. DOI: 10.1107/S1600576724007659/dv5017sup4.mp4

Video Example STRUCTURE RELATIONS: related to the example in section 6 that shows how to use the program STRUCTURE RELATIONS. DOI: 10.1107/S1600576724007659/dv5017sup5.mp4

Video THIOUR01 to 02 transformation VESTA: related to the example in section 6 that shows how to transform the structures using VESTA. DOI: 10.1107/S1600576724007659/dv5017sup6.mp4

Video Example SETSTRU: showing how to transform structures using the program SETSTRU with the examples from Section 6. DOI: 10.1107/S1600576724007659/dv5017sup7.mp4

Video Example TRANSTRU: showing how to transform structures using the program TRANSTRU with the examples from Section 6. DOI: 10.1107/S1600576724007659/dv5017sup8.mp4

Video Example COMPSTRU: showing how to compare structures using the program COMPSTRU with the examples from Section 6. DOI: 10.1107/S1600576724007659/dv5017sup9.mp4

## Figures and Tables

**Table 1:** *Jmol*load command options relating to crystallographic symmetry The reader can experiment with the *Jmol* commands in this table at https://chemapps.stolaf.edu/jmol/jsmol/iucrdemo. Further details of this resource are given in the supporting information (file 2).

Option	Action	Example
<n>	Load just the third model	load =ams/quartz 3;
{na nb nc}	Create an *na* × *nb* × *nc* block of unit cells, ‘packing’ the cells with atoms on all faces	load =ams/quartz 1 {2 2 2};
packed [range]	Add packing atoms on all faces, or optionally a range in fractional units beyond the faces	load =aflowlib/123 packed
		load =ams/quartz 1 packed 0.6
SUPERCELL <description>	Fill a supercell of the designated size, producing a single larger cell	load =aflowlib/12.5 SUPERCELL {2 2 1}
		load =ams/quartz 1 supercell "2a,a+2b,c"
fill <x>	Fill an *x* by *x* by *x* Å block of space with atoms, regardless of the unit cell	load =ams/halite 1 fill 20
fill unitcell primitive	Used to reload the current file, filling its primitive unit cell rather than its conventional cell	load =aflowlib/166.51;
		load "" fill unitcell primitive
spacegroup "x,y,z;x,-y,z"	Load a file, overriding its space group with the one provided or, as in this case, with only the specified two operations, effectively loading the file using space group 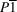	load =aflowlib/62.3 spacegroup "x,y,z;x,-y,z"
spacegroup <space group> unitcell <unit-cell definition> offset <offset>	Apply the specified space group, unit cell and offset to the file data, which need not be crystallographic; this example loads caffeine from PubChem, shifting the data 2 Å in the *z* direction, and then applies the symmetry of the *P*2_1_ space group (No. 4)	load :caffeine centroid spacegroup "P21" unitcell [10 10 10 90 90 90] offset {0 0 2}

**Table 2:** *Jmol*modelkit commands relating to crystallography The reader can experiment with the *Jmol* commands in this table at https://chemapps.stolaf.edu/jmol/jsmol/iucrdemo.

*Jmol* command	Action	Example
modelkit spacegroup <name or number>	Creates or changes the space group to the given name or number	zap; modelkit spacegroup 62
		modelkit spacegroup "Pnma"
		modelkit spacegroup "ITA/62.1"
modelkit add <element> <position> [packed]	Add a set of equivalent atoms in the specified element on the position, expressed as a Cartesian or fractional coordinate, or a Wyckoff position, including *G* for ‘general’. For atoms on faces, packing is optional	modelkit add O {1/4,1/2,1/4}
		modelkit add N {0.12,0.23,0.45/1}
		modelkit add N wyckoff G
		modelkit add O {1/2,1/2,0} packed
modelkit moveto <atom> <coordinate>	Move an atom to a new position, also adjusting its symmetry-equivalent atoms appropriately	modelkit moveto @3 {0.27 0.32 0.45/1}
modelkit assign <elem>	Changes the element of the specified atom and its equivalent atoms	modelkit assign @3 Ge
modelkit connect <atom1> <atom2>	Connects the two specified atoms, and their equivalent atoms accordingly	modelkit connect @4 @5
modelkit delete <atom1>	Deletes the specified atom or set of atoms and all of their equivalent atoms	modelkit delete @3
		modelkit delete _Mn
		modelkit delete {wyckoff=c}
		modeklit delete {site=2}
		modelkit delete {fz > 0.9}
draw spacegroup [ALL]	Draws the symmetry elements of a space group, either just the minimal representation set, or the full set of operations that are typically shown in a space-group symmetry diagram	zap; modelkit 62; draw spacegroup
		load =aflowlib/62.1; draw spacegroup ALL
draw symop …	Depicts a specific symmetry operation associating one atom or coordinate with another	draw symop 3
		draw symop @3 @4
		draw symop {0 1/4 0} {0 3/4 0}

**Table 3:** Various questions and how to answer them using *Jmol* scripting functions The reader can experiment with the *Jmol* commands in this table at https://chemapps.stolaf.edu/jmol/jsmol/iucrdemo.

Question	*Jmol* script	Answer
What is an example of a structure with *Pmna* symmetry?	print spacegroup("pnma").ita	62
	load =aflowlib/62 packed	Structure loads, and *Jmol* reports: TITLE: Magnetic and Structural Properties of Transition Metal Substituted MnP
What are the Wyckoff positions and coordinates for space group 62?	zap; modelkit spacegroup 62; print symop("wyckoff")	*a* (0, 0, 0) (1/2, 0, 1/2) (0, 1/2, 0) (1/2, 1/2, 1/2)
		*b* (0, 0, 1/2) (1/2, 0, 0) (0, 1/2, 1/2) (1/2, 1/2, 0)
		*c* (*x*, 1/4, *z*) … (three more)
		*d* (*x*, *y*, *z*) … (seven more)
What is the general position in space group 225?	zap; modelkit spacegroup 225; print symop("wyckoff", "G")	l (*x*, *y*, *z*) (−*x*, −*y*, *z*) … (190 more)
What are the representative symmetry operations for space group 145?	show spacegroup 145	Three operators from *P*32 (No. 145): 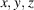 ; 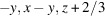 ; 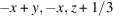
What are the generators for the first setting of space group 100?	print spacegroup(100).its[1].gen	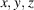 ; 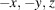 ; 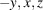 ; 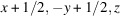
Which space groups have rhombohedral settings?	print spacegroup("ita/all").select("(sg) where hm like ’*:r’").join(",")	146, 148, 155, 160, 161, 166, 167
What are the Wyckoff positions of the copper atoms in this structure?	load =aflowlib/220.3 packed; print {_Cu}.wyckoffm.pivot.format("JSON")	{"12*a*": 18, "48*e*": 48}

**Table 4:** Representative websites incorporating *JSmol* in the area of crystallography

Site	Use
Bilbao Crystallographic Server *COMPSTRU* program	A program for the comparison of crystal structures
Crystallography Open Database	Displaying interactive previews of small-molecule crystal structures
American Mineralogist Crystal Structure Database	Simple interactive display of mineral structures
AFLOW Encyclopaedia of Crystallographic Prototypes	A catalogue of crystal structures spanning the full range of space groups
IUCr Journals†	Interactive 3D views of crystal structures related to both limited and open access publications
Q-Studio Crystal Structure Builder‡	A functionally rich site for the menu-driven construction of crystal structures using *JSmol*, especially designed for computational structure creation
*Jmol* Crystal Symmetry Explorer§	Allows the exploration of the relationship between symmetry operations and their associated geometric elements
*Jmol *Space Group Symmetry Visualizer¶	Allows detailed exploration of space-group symmetry, including Wyckoff positions, starting from either general space-group models, a wide range of curated models or models provided by the page visitor
*Jmol* Point Group Explorer††	Test yourself on your ability to determine the point group of a small molecule with this simple webpage
IUCr Symmetry Workshop Demo‡‡	A webpage designed specifically for this article, allowing readers to experiment for themselves with the various scripting capabilities of *Jmol*

† https://publcif.iucr.org/services/tools/.

‡ https://qs.pwmat.com.

§ https://chemapps.stolaf.edu/jmol/jsmol/jcse.

¶ https://spacegroups.symotter.org.

†† https://chemapps.stolaf.edu/jmol/jsmol/jpge.

‡‡ https://chemapps.stolaf.edu/jmol/jsmol/iucrdemo.

## Footnotes

1 The conventional settings of space groups are those fully described in *IT*A. This means that the corresponding data, including diagrams, asymmetric units, generators, and general and special Wyckoff positions, can be found in *IT*A.

2 In the BCS we refer to all those settings that are listed in the synoptic tables of plane and space groups found in ch. 1.5 of *IT*A as *IT*A settings. For us the *Pnma* setting is the conventional (and standard) setting of the space group *Pnma* (No. 62), while *Pmnb*, *Pbnm*, *Pcmn*, *Pmcn* and *Pnam* are just *IT*A settings of *Pnma*.

3 We have selected this small organic molecule for many reasons. The phase transition described in this section is accompanied by a simple to understand structure–property correlation. The molecule is small enough and the symmetry simple enough (without being obvious) to allow performing calculations by hand, and it is a great first step to enter into more complex structures (*e.g.* incommensurate phases). Finally, it is a cheap to purchase chemical that is very easy to recrystallize and shows its phase transitions in a temperature range accessible to most in-house laboratories, allowing the transformation of the analysis in this paper into a practical session.

4 The four thiourea entries from the CSD discussed in this paper are available in the free Teaching Subset of the CSD.

5 *P*2_1_*ma* is an *IT*A setting of space group *Pmc*2_1_ (No. 26) obtained by renaming the *abc* axes to *cab* (see Table 1.5.1.1 of *IT*A for transformation matrices). The *P*2_1_*ma* setting of thiourea can be converted to the standard *Pmc*2_1_ with *VESTA* using the *Edit/Edit Data/Unit Cell* tab, selecting setting number 1 *Pmc*2_1_ (**a**, **b**, **c**) and clicking the *Apply* button.

6 *Pmc*2_1_ is a maximal subgroup of index 2 of *Pnma*, as could be confirmed using the *MAXSUB* program in the BCS (see Section 2.2.1). In particular it is a *translationengleiche* subgroup, since translations are kept but the order of the point group is reduced.

7 Conversion from *Pbnm* to *Pnma* can be performed in *VESTA* using the *Edit/Edit Data/Unit Cell* tab, selecting setting number 1, *Pnma* (**a**, **b**, **c**), and clicking the *Apply* button; in *Jmol* it can be done with the command modelkit spacegroup "Pnma".

8 The tripled cell with *Pnma* symmetry is a *klassengleiche* subgroup of the original ambient-pressure cell with the same space-group symmetry.
