# Supplementary material for: Free tools for crystallographic symmetry handling and visualization
Source: J Appl Crystallogr. 2024 Sep 20;57(Pt 5):1618–39. doi: 10.1107/S1600576724007659 (PMC11460394; doi:10.1107/S1600576724007659)
Supplement: Supplementary file 2 [file j-57-01618-sup2.pdf]

JOURNAL OF
APPLIED
CRYSTALLOGRAPHY

Volume 57 (2024)

Supporting information for article:

Free tools for crystallographic symmetry handling and visualization

Gemma de la Flor, Mois I. Aroyo, Ilaria Gimondi, Suzanna C. Ward, Koichi Momma, Robert M. Hanson and Leopoldo Suescun

Web-based visualization of crystal structure and symmetry using Jmol

Supporting Information, by Bob Hanson hansonr@stolaf.edu

Supporting information for the article, *Online tools for crystallographic symmetry handling and Visualization* specifically relating to Jmol can be opened by any standard browser at https://chemapps.stolaf.edu/jmol/jsmol/iucrdemo

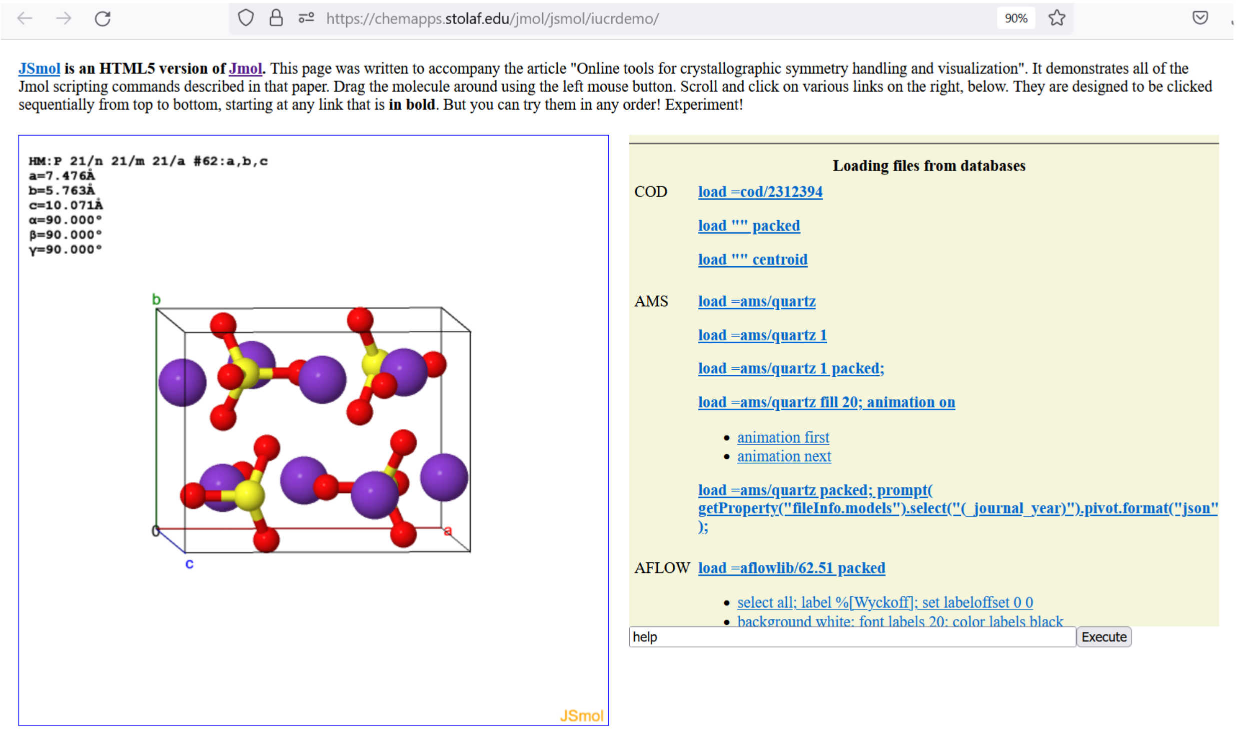

The page includes three elements:

- a JSmol window
- a scrollable table of commands that, when clicked, operate on the window
- a command-line input box (seeded with the “help” command)

Running the commands

At this page you can run all the commands discussed in the paper, particularly in Table 1-3, and see their result. In addition, the sequences of commands used to produce the models for Figures 15-17 are given. Using these, you can create (and save!) those images yourself.

Instructions at the top of the page read:

> *Drag the molecule around using the left mouse button. Scroll and click on various links on the right, below. They are designed to be clicked sequentially from top to bottom, starting at any link that is **in bold**. But you can try them in any order! Experiment!*

In addition, below the scrolling table, there is Jmol Script input box. You can enter any valid Jmol command (including ***help*** to see command documentation) or sequence of Jmol commands into that box. For example, you could enter the following to see a “movie” of all 230 space group symmetry models:

for (var i=1;i<=230;i++){modelkit draw spacegroup @i;refresh}

(and enter ***!quit***) to stop it, if you want to. Or you could pull up a Jmol scripting console by entering:

console

which would allow you to enter commands and also see their results, if they provide information (such as ***show symops*** or ***print spacegroup(110)***.

You can also open a pop-up context menu by right-clicking in the JSmol window. Many additional actions are available in this way.

Saving models

Using the Jmol ***write*** command or the context menu item **File…Export…** you can save the model or model images. Note that all of the PNG files created by Jmol/Java or JSmol from using the MODELKIT command, and all extended PNG files ("PNGJ" format) that you create from models generated starting with **LOAD** can be drag-dropped or otherwise loaded into Jmol/Java or JSmol to recreate the model in the exact state it was in when you saved it.

Jmol 16.2.11 was used in the creation of the models for this article. Earlier versions may not give the same results.

Creating your own webpage

It is exceptionally easy to create a web page with a JSmol window and active links to "drive" it. Look behind the scenes, and you will see that this web page is just a template. It loads a couple of JavaScript files

```
<script type="text/javascript" src="../JSmol.min.js"></script>

<script type="text/javascript" src="code.js"></script>
```

The first of these, JSmol.min.js, initializes JSmol.

The second provides the information that we see in the table (the contents of "demodiv").

And it indicates where on the page to put the three elements:

```
<td valign="top">

   <div id="appdiv"></div>

</td>

<td valign=top>

   <div id=demodiv style="background:beige;width:600px;height:500px;overflow:auto"></div>

   <div id=cmddiv></div>

</td>
```

An additional bit of boilerplate scripting installs Jsmol. (View the page source for that)

If you are interested in creating web pages, I encourage you to contact me at hansonr@stolaf.edu or check out the Jmol Users List, which is quite active and very responsive. You can subscribe to the list at https://sourceforge.net/p/jmol/mailman/jmol-users/ (see the link just above the table)

Jmol source and distribution

This web page is part of a larger Jmol distribution package available for download at https://sourceforge.net/projects/jmol/files/ as Jmol-<version>-binary.zip. Unzipping that file, you will find jsmol.zip. And unzipping that, you will find the jsmol/iucrdemo directory.

Jmol is an open-source Eclipse project hosted at SourceForge (https://sourceforge.net/projects/jmol). As such, you can import the project into Eclipse and build it there. Part of the build includes automatically "transpiling" the Jmol source code to JavaScript. If you are interested in contributing to the Jmol project, please contact me directly.

Bob Hanson, 2024-04-29

hansonr@stolaf.edu
